# Supplementary material for: Systematically assessing microbiome–disease associations identifies drivers of inconsistency in metagenomic research
Source: PLoS Biol. 2022 Mar 2;20(3):e3001556. doi: 10.1371/journal.pbio.3001556 (PMC8890741; doi:10.1371/journal.pbio.3001556)
Supplement: S1 Fig — (A–C) The impact of the different numbers of vibrations and data transformation methods on VoE. We plot the number of features that were FDR significant at least once upon vibration with different numbers of adjusting variables considered as well as different data transformation strategies (i.e., logged versus raw abundances versus center log ratio transformations). (D) The number of p-value and (E) FDR significant findings for the T2D cohort with the largest number of possible adjusters (using only log-transformed data, as opposed to the previous 3 panels). (F) The robustness of associations as a function of number of vibration variables and modeling strategy. We computed the fraction of associations that were positive for any given microbial feature—a highly robust association is 100% positive or 0% positive (i.e., negative), whereas a nonrobust association is closer to 50% positive (i.e., inconsistent in direction). In this heatmap, we correlated these associations for all features to gauge if the different data transformations and numbers of adjusting variables yielded similar measures of robustness across all datasets. This figure can be generated using the code deposited in https://github.com/chiragjp/ubiome_robustness and the data deposited in https://figshare.com/projects/Microbiome_robustness/127607. FDR, false discovery rate; T2D, type 2 diabetes; VoE, vibration of effects. (PDF) [file pbio.3001556.s003.pdf]

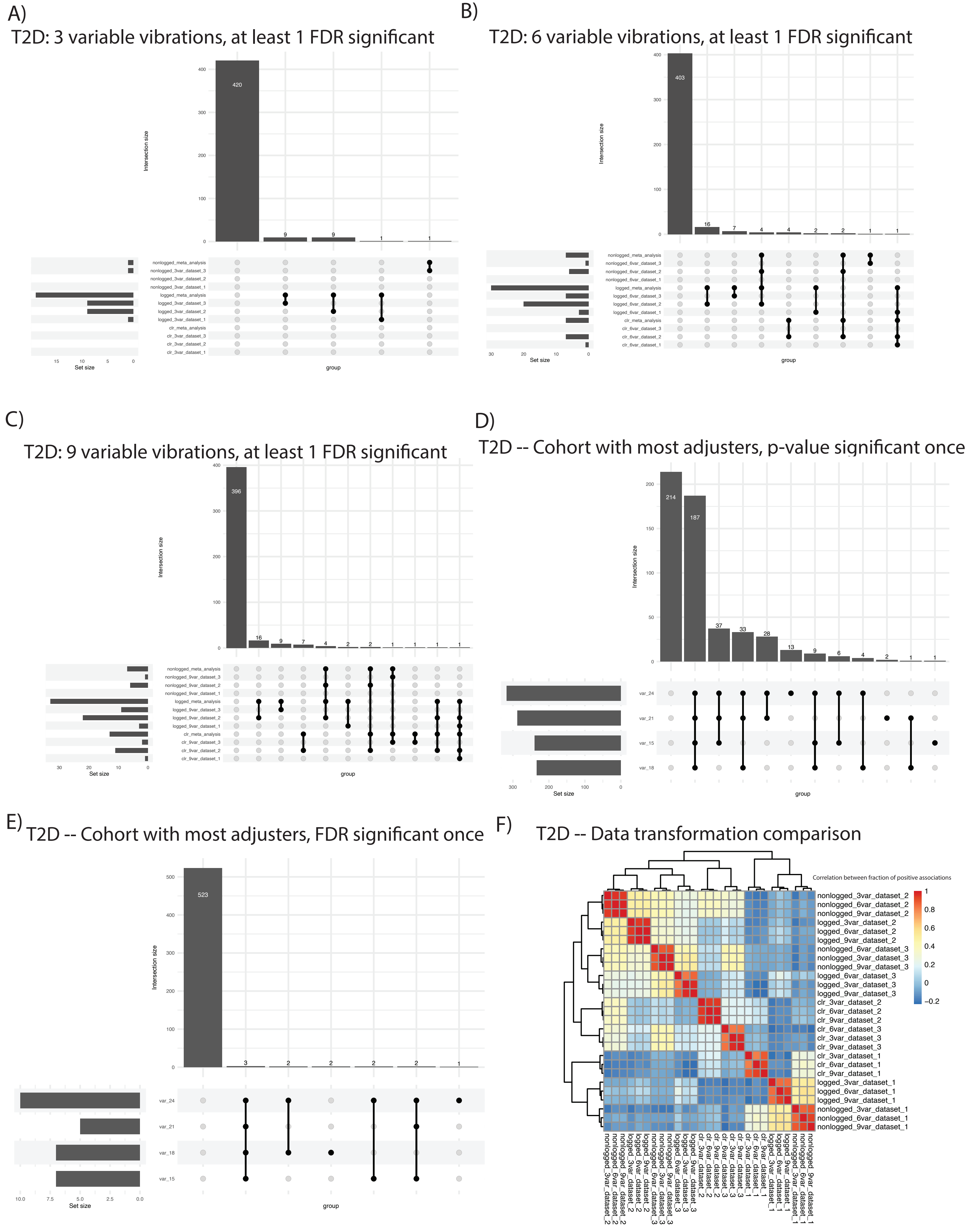

A)
T2D: 3 variable vibrations, at least 1 FDR significant
Intersection size
420
9
9
1
1
nonlogged_meta_analysis
nonlogged_3var_dataset_3
nonlogged_3var_dataset_2
nonlogged_3var_dataset_1
logged_meta_analysis
logged_3var_dataset_3
logged_3var_dataset_2
logged_3var_dataset_1
clr_meta_analysis
clr_3var_dataset_3
clr_3var_dataset_2
clr_3var_dataset_1
Set size
group
B)
T2D: 6 variable vibrations, at least 1 FDR significant
Intersection size
403
16
7
4
4
2
2
1
1
nonlogged_meta_analysis
nonlogged_6var_dataset_3
nonlogged_6var_dataset_2
nonlogged_6var_dataset_1
logged_meta_analysis
logged_6var_dataset_3
logged_6var_dataset_2
logged_6var_dataset_1
clr_meta_analysis
clr_6var_dataset_3
clr_6var_dataset_2
clr_6var_dataset_1
Set size
group
C)
T2D: 9 variable vibrations, at least 1 FDR significant
Intersection size
396
16
9
7
4
2
2
1
1
1
1
nonlogged_meta_analysis
nonlogged_9var_dataset_3
nonlogged_9var_dataset_2
nonlogged_9var_dataset_1
logged_meta_analysis
logged_9var_dataset_3
logged_9var_dataset_2
logged_9var_dataset_1
clr_meta_analysis
clr_9var_dataset_3
clr_9var_dataset_2
clr_9var_dataset_1
Set size
group
D)
T2D -- Cohort with most adjusters, p-value significant once
Intersection size
214
187
37
33
28
13
9
6
4
2
1
1
var_24
var_21
var_15
var_18
Set size
group
E)
T2D -- Cohort with most adjusters, FDR significant once
Intersection size
523
3
2
2
2
2
1
var_24
var_21
var_18
var_15
Set size
group
F)
T2D -- Data transformation comparison
Correlation between fraction of positive associations
nonlogged_3var_dataset_2
nonlogged_6var_dataset_2
nonlogged_9var_dataset_2
logged_3var_dataset_2
logged_6var_dataset_2
logged_9var_dataset_2
nonlogged_6var_dataset_3
nonlogged_3var_dataset_3
nonlogged_9var_dataset_3
logged_6var_dataset_3
logged_3var_dataset_3
logged_9var_dataset_3
clr_3var_dataset_2
clr_6var_dataset_2
clr_9var_dataset_2
clr_6var_dataset_3
clr_3var_dataset_3
clr_9var_dataset_3
clr_3var_dataset_1
clr_6var_dataset_1
clr_9var_dataset_1
logged_3var_dataset_1
logged_6var_dataset_1
logged_9var_dataset_1
nonlogged_3var_dataset_1
nonlogged_6var_dataset_1
nonlogged_9var_dataset_1
