# Supplementary figures and images for: Systematically assessing microbiome–disease associations identifies drivers of inconsistency in metagenomic research
Source: PLoS Biol. 2022 Mar 2;20(3):e3001556. doi: 10.1371/journal.pbio.3001556 (PMC8890741; doi:10.1371/journal.pbio.3001556)

## ACVD

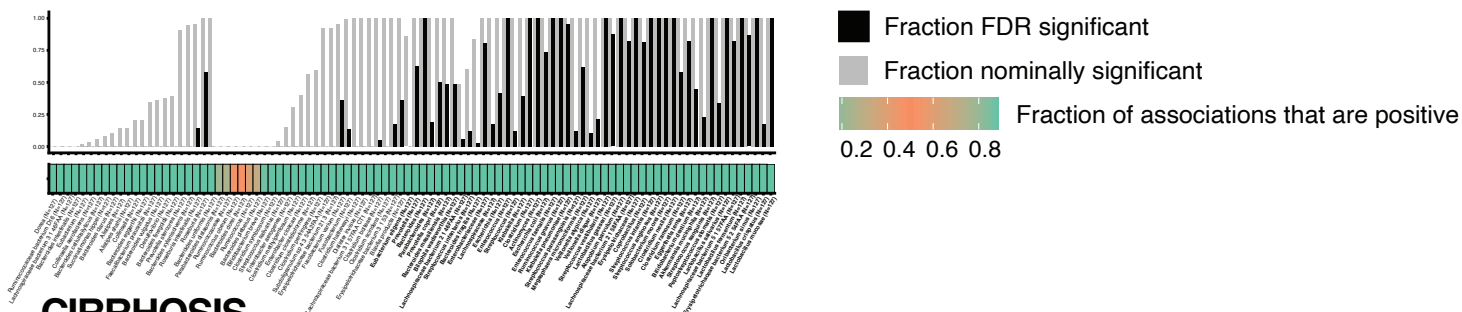

## CIRRHOSIS

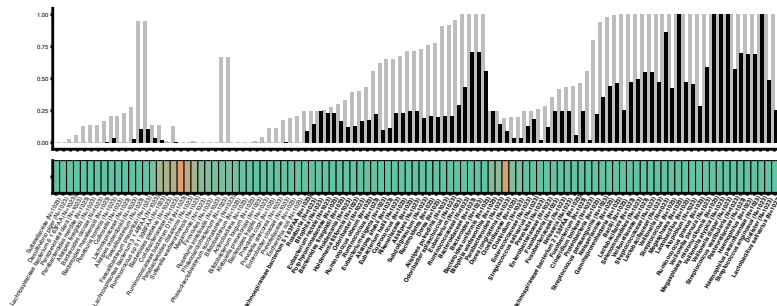

## CRC

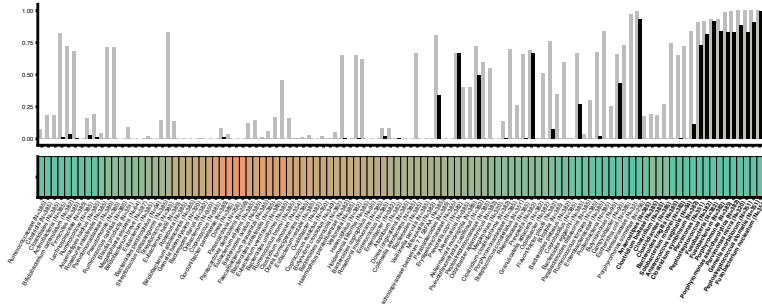

## IBD

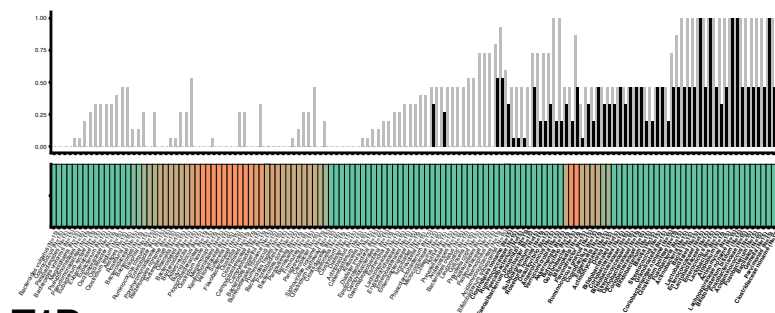

## T1D

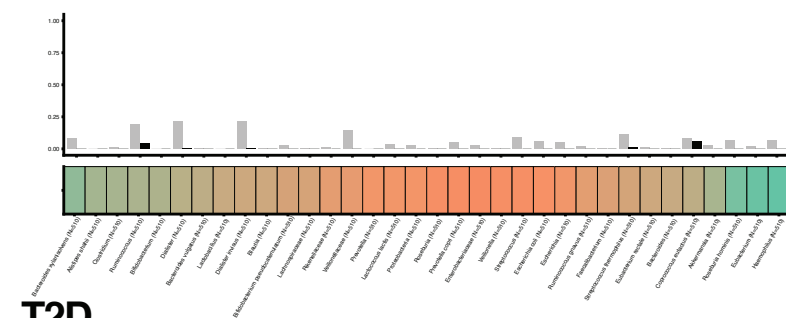

## T2D

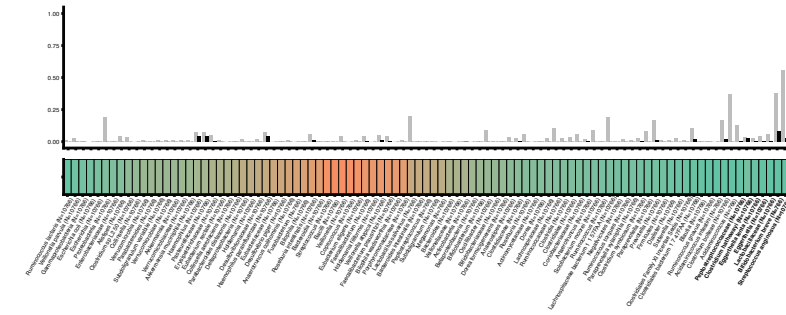

Supplement: S2 Fig — The middle bar describes the fraction of association sizes greater than 0 for a given association: A highly confounded association will be closer to 0.5 and pink, whereas more robust associations will be closer to 0 or 1 and blue. The gray bars in upper bar plot corresponds the fraction of models that were nominally (p-value < 0.05) significant for the microbial feature–disease association, whereas the black bars correspond to the fraction of models that were FDR significant. This figure can be generated using the code deposited in https://github.com/chiragjp/ubiome_robustness and the data deposited in https://figshare.com/projects/Microbiome_robustness/127607. FDR, false discovery rate; VoE, vibration of effects. (PDF) [file pbio.3001556.s004.pdf]

*Roseburia* and ACVD

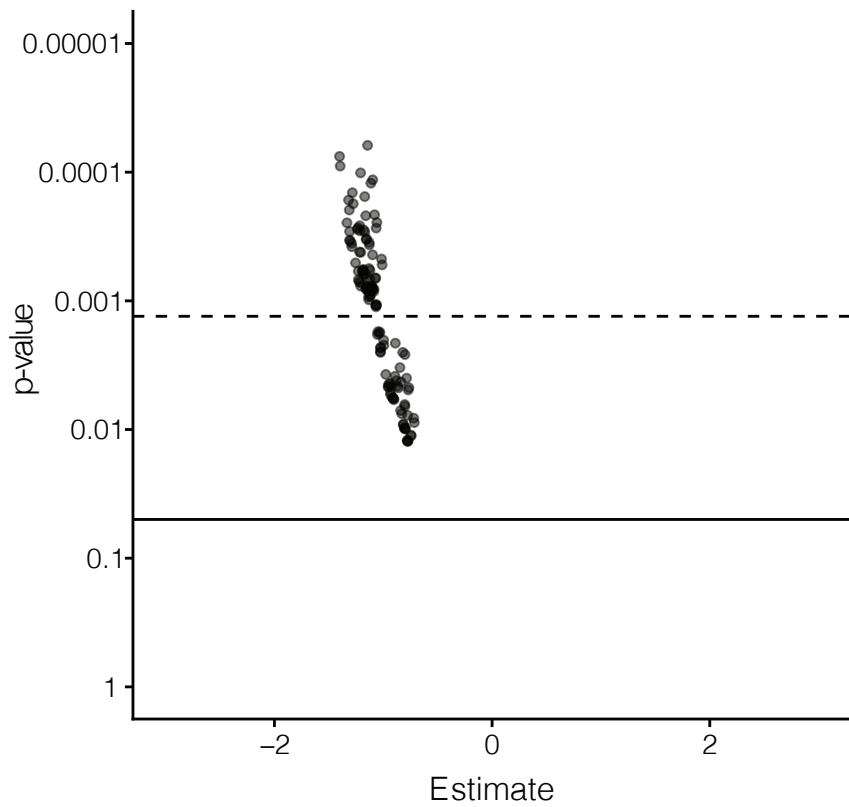

Supplement: S3 Fig — Each point represents a different model specification, the x-axis is the beta coefficient on the binary disease variable, the y-axis is the −log10(p-value). The dotted line represents FDR adjusted significance. The solid line represents nominal significance. This figure can be generated using the code deposited in https://github.com/chiragjp/ubiome_robustness and the data deposited in https://figshare.com/projects/Microbiome_robustness/127607. ACVD, atherosclerotic cardiovascular disease; FDR, false discovery rate. (PDF) [file pbio.3001556.s005.pdf]

A)

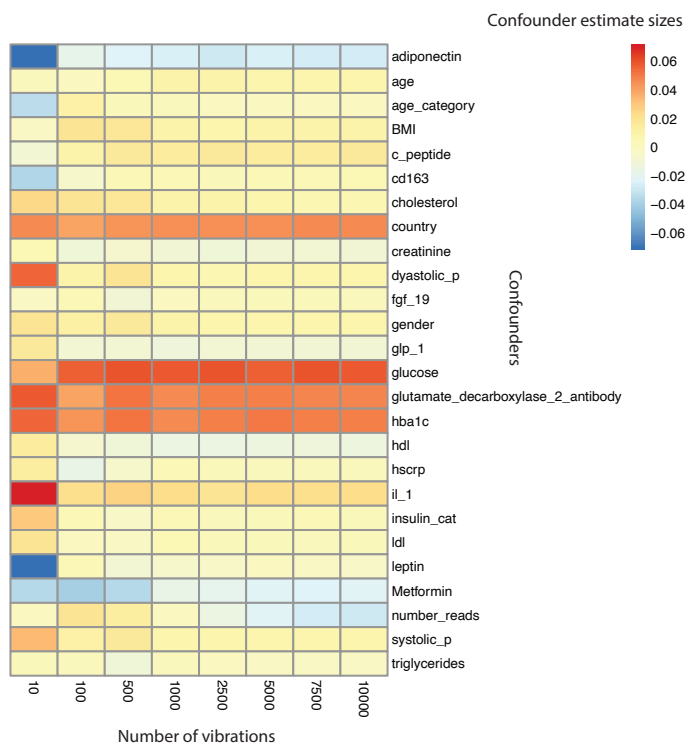

B)

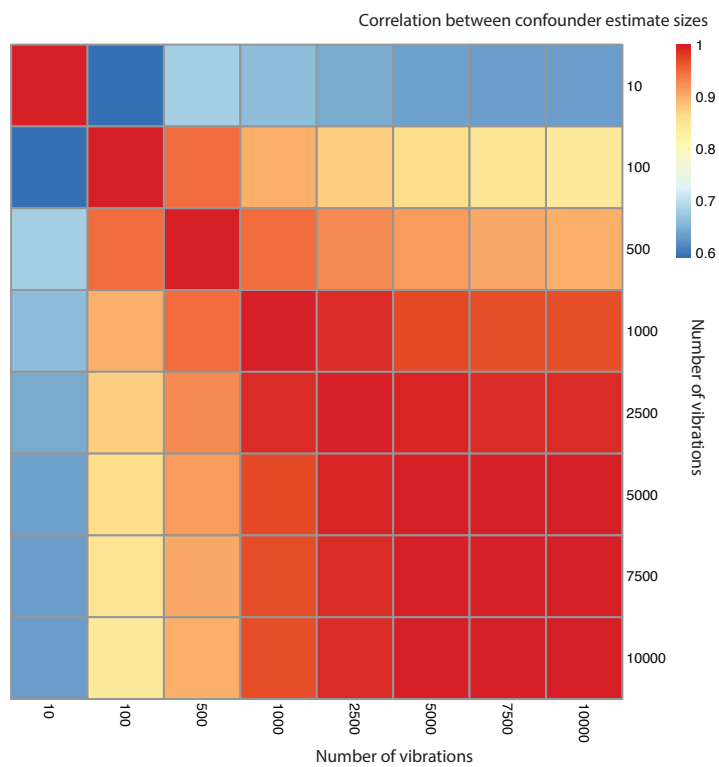

Supplement: S4 Fig — (A) The output of our confounder analysis (e.g., in Fig 4). The x-axis is the number of vibrations. The y-axis is each possible adjusting variable in the T2D associations. The values correspond to the beta coefficient (from our mixed effects analysis) describing the average change in microbiome–disease associations when a given adjusting variable is present in a model. (B) The correlation between the values in panel A at different numbers of vibrations. This figure can be generated using the code deposited in https://github.com/chiragjp/ubiome_robustness and the data deposited in https://figshare.com/projects/Microbiome_robustness/127607. T2D, type 2 diabetes. (PDF) [file pbio.3001556.s006.pdf]
