# Supplementary material for: Systematically assessing microbiome–disease associations identifies drivers of inconsistency in metagenomic research
Source: PLoS Biol. 2022 Mar 2;20(3):e3001556. doi: 10.1371/journal.pbio.3001556 (PMC8890741; doi:10.1371/journal.pbio.3001556)
Supplement: S1 Text — (DOCX) [file pbio.3001556.s007.docx]

##### The impact of the number of adjusting variables and data transformation method on VoE

We additionally benchmarked additional aspects of our VoE approach for our T2D cohorts, which contained the largest number of adjusting variables in our dataset. We compared different data transformations prior to modeling (centered-log-ratio [CLR] vs. log-transformed vs. unmodified abundance values) as well as vibrations with different numbers of variables. Different transformation methods yielded different results: log-transforming variables resulted in the largest number of associations that were at least FDR significant once (Supp Fig 1A-C). The number of vibration variables also slightly changed output, with increasing number of adjusters (and therefore vibrations) yielding more taxa, as one would expect that were FDR-significant at least once, indicating a potential drawback of misuse of VoE. That said, between vibrating over 3, 6, and 9 variables (the number of variables in the cohort with the fewest adjusters), the number of potentially significant features for meta-analyzed, log-transformed datasets went from 19 to 30 to 31.

We additionally executed a similar, non-meta-analytic, analysis in the T2D cohort with the largest number of potential adjusters (24 variables with natural logged data only, Supp Fig 1D-E). We observed consistency in the number of FDR significant variables and those that were p-value significant at least once, for the most part, across different numbers of adjusters. There was some variation however, with the number of ostensibly significant features (especially in the p-value-significant-once category, Supp Fig 1D) slightly increasing as a function of number of adjusters, indicating the potential for excessive vibrations and reliance on p-values alone to yield increased false positives (or, conversely, at the very least, more associations worth investigating).

Finally, we estimated changes in ostensible robustness of different associations for the three data transformation strategies and number of variables vibrated over (Supp Fig 1F). For all taxa, estimated the correlation between the fraction of all associations for a given taxon that were positive (a measure of how consistent, or robust, an association is). Highly robust associations have entirely positive (fraction approaching 1) or entirely negative (fraction approaching 0) association signs. Non-robust associations have fractions closer to 0.5 (i.e. 50% of models producing conflicting results. We found high correlations (>.9) between these values for given transformation methods regardless of the number of vibrations selected. In other words, a robust association was consistent regardless of the number of vibration variables selected.
