# Supplementary material for: Systematically assessing microbiome–disease associations identifies drivers of inconsistency in metagenomic research
Source: PLoS Biol. 2022 Mar 2;20(3):e3001556. doi: 10.1371/journal.pbio.3001556 (PMC8890741; doi:10.1371/journal.pbio.3001556)
Supplement: Attachment — Submitted filename: robustness_response_to_reviewers.pdf [file pbio.3001556.s008.pdf]

To the Editors and Reviewers,

Thank you for your consideration of our manuscript, and we thank you for your patience as we prepared these revisions. We are grateful for the time you took to review and provide constructive comments. We have integrated your comments into our work and find it to be much improved. We are enclosing here and updated manuscript in the hopes that you will feel the same.

Overall, we made the following broad changes:

1) We clarify the intent of the paper, which is not to focus on explicitly on reproducibility but rather primarily to compare fitting a single model vs many different ones via VoE as a measure of robustness. This additionally involved clarifying our methods regarding our literature review and (lack of) any *a priori* hypotheses.
2) We reworked the first half of the manuscript to focus on all associations in our dataset instead of just those in the literature. We integrated the points of Reviewer 1 and Reviewer 2 and chose to separate Figure 1 in to 2 new figures, reorder the panels, and perform new analyses that are clearer in their intent to demonstrate the utility of modeling VoE for the microbiome.
3) We justify the use of VoE and carry out extensive benchmarking analyses where we systematically compare the numbers of adjusting variables and data transformation strategy on model output.

In this document, we provide a point-by-point response to Reviewer comments in blue. We additionally reproduce passages from the revised manuscript, highlighting particularly key changes in yellow. We additionally provide both a clean, updated manuscript as well as one with changes tracked.

Once more, thank you for your effort and time, and we look forward to your response.

Sincerely, and on behalf of the authors,

Chirag J Patel and Aleksandar D Kostic

Reviewer #1:

I think that this manuscript has the potential to become an important contribution to the field. I have however mixed feelings about some aspects that I will try to detail in my review.

Please note that I am also reviewer for another paper of this team in PLOS Biology and that I was also very enthusiastic about this other paper. Please also note that I am a reviewer involved in meta-research and that I strongly suggest that a reviewer involved in the specific field of microbiome research should be invited (a reviewer used to the methods used in this field).

First of all, this is a stellar piece of research performing various "case studies", applying the concept of vibration of effects in the field of gut microbiome disease associations. VoE is a form of multiverse analysis and is the subject of many research efforts across the world and the team submitting this paper is at the forefront of these efforts. The microbiome field is rapidly expanding and may have serious problem of reproduciblity due to somewhat small sample size and an universe of researcher's degrees of freedom. The application of VoE concept in this case is therefore timely and innovative. One may also note that VoE is proposed here in a very positive manner, as a tool to identify associations worth of interest and not as a "brute force" tool to disqualify any positive association. This paper represent an heroic effort not solely in running various models but also in being an attempt to synthesize certain aspects the field of microbiome research. It is, in my opinion an important contribution to the field of meta-research.

We are delighted that the Reviewer identified so succinctly our goal in preparing this manuscript regarding the identification of new associations of interest, and we are extremely grateful for their kind words.

I however have two major concerns :

1.1) First the research was not pre-registered (or perhaps I missed it). Of course, authors may acknowledge that this is not an hypothesis generating study. But, in my opinion, the paper presents many specific examples as cases studies and there is room for cheery picking. I don't say that authors cherry picked the results but, rather that, without pre-registration, one cannot affirm that it was not the case

Several choices were made in choosing the examples/case studies. Pre registration would have added more transparency on these choices

I regret that the authors didn't adopt a systematic review approach. It could have helped again in providing more transparency concerning selection of the included topics / case studies ;

Some aspect of the systematic review approach may have been useful to improve both reporting and reproducibility (e.g. a PRISMA flow chart may have been much more intuitive and easy to follow that the current information reported in the various web appendices)

I anticipate that authors will say that registration may not be mandatory because this is not an hypothesis testing study, and I can understand this point of view. But I also think that there are some degrees of freedom in their study and that it is always best to fix those a priori, as far as it is possible of course ;

At the very least, non-registration must be explicitly stated in the methods / and / possible shortcomings must be discussed in the limitations ;

We agree with the Reviewer that pre-registration is a good idea for hypothesis generation, however they have correctly identified the major reason why we did not do so initially: that being that this study was not associated with hypothesis testing and instead falls more in line with hypothesis generation. This critique, however, is warranted, and while we do not doubt the soundness of our methods we sympathize with the Reviewer's point. We additionally would like to note that pre-registration is not common in microbiome studies in general -- this is clearly another issue with the field as a whole, as it is a standard that should be set (at least in hypothesis testing).

As requested, we now explicitly state in the methods that the study was not pre-registered. We additionally describe limitations therein. Similarly, we additionally discuss (in the same region of the Discussion) our review strategy, which as the reviewer mentioned was not systematic.

We write:

"We would additionally like to note that our study was not pre-registered. While this is typical for both microbiome studies and hypothesis generating (vs. hypothesis testing) studies in general, we do acknowledge (and we thank a reviewer of this manuscript for pointing this out) that it would be useful for these meta-science applications going forward. Indeed, given that our literature review involved searching for single reports of microbial species already in our dataset associated with disease (as opposed to looking for all possible reported associations), some form of pre-registration could have clarified this approach. Going forward pre-registration would set an even higher bar for robust MAS."

We additionally write in the drawbacks and limitations section of the Discussion:

"Of course, our approach is not without other drawbacks. First, we did not pre-register our study, which, would set an even higher bar for reproducible results."

If the editors or Reviewer have any other recommendations to deal with specific degrees of freedom in question, we will happily oblige.

It seems that the authors used a protocol (p.13, l.377) but they do not provide this protocol / I would be interested in a specific pargraph detaillin any change to the protocol;

We apologize for the lack of clarity here -- the sentence in question, which stands separated by whitespace from the rest of the manuscript at l. 377 reads

“We had additionally specific sub-protocols for the phenotypes in certain cases.”

This, of course, appears to be out of place and unclear. However, in the following four blocks of text, we describe the phenotype-specific protocols we implemented. To clarify that this sentence refers to these protocols, we have indented the blocks of text in question and also now write:

“We had additionally specific sub-protocols for the phenotypes in certain cases, which we detail in the following four blocks of text.”

1.3) My second major concern is rather conceptual:

Authors suggest that their approach is an advance in identifying association worth of interest. At least this is the tone of the discussion and the conclusion. I do think that there is some spin here and that the design cannot garantee this.

We agree with this comment, and Reviewer 2 raised a similar point regarding the tone of our discussion and the lack of a clear “association prioritization framework.” We have endeavoured to make our language both more specific and less hyperbolic with regards to these critiques, and we hope the Reviewers will find it much improved and clearer. Here are passages that we focused on in particular:

In the final paragraph of the Introduction, where we set up the aims for the paper, we now write:

“Here, to gauge the impact of model specification in MAS, we deploy a systematic sensitivity analysis, measuring Vibration of Effects in reported microbiome associations. Comparing modeling strategies, we quantify the robustness(variation as a function of model specification) in microbial taxon-disease associations across six different phenotypes. With an emphasis on 581 associations that were reported in the literature, we counted how many associations (published and otherwise) are recovered (e.g. appear as statistically significant) when undergoing sensitivity analysis. We propose modeling VoE as one of many potential steps in building association prioritization frameworks, metrics for prioritizing microbiome findings for *in vivo* validation.”

And in the Discussion, we now write:

“We claim that one step -- out of many -- towards translating microbiome findings into biological understanding is determining how best to prioritize for future (e.g. *in vivo*) investigation associations arising from MAS.”

“That said, modeling VoE is certainly not the only way to identify an association worth prioritizing. Furthermore, an ostensibly robust association viewed only through the lens of VoE

still could be a false positive or dependent on, for example, data processing pipeline choice (e.g. the decision to average repeated measures data vs. selecting one sample per individual). A more comprehensive framework could rely on a number of heuristics, for example putting the greatest emphasis on associations that have the best model fit, are reported across multiple large cohorts, and/or have undergone sensitivity analysis via VoE."

Indeed the present study nicely details a path for identifying associations that can be considered as robust regarding various criteria ;

This was one of the core aims of our study, and we are grateful the Reviewer felt it was achieved.

But it does not mean that the robust associations identified will necessarily be transformed in more robust discoveries ;

We found this point to be extremely insightful and have integrated it into our discussion. We feel that while a robust association (as identified via VoE) is necessary for a robust discovery, we agree with the Reviewer that it may not be sufficient on its own. As a result, we now write in the Discussion (we include this passage above as well):

"That said, modeling VoE is certainly not the only way to identify an association worth prioritizing. Furthermore, an ostensibly robust association viewed only through the lens of VoE still could be a false positive or dependent on, for example, data processing pipeline choice (e.g. the decision to average repeated measures data vs. selecting one sample per individual). A more comprehensive framework could rely on a number of heuristics, for example putting the greatest emphasis on associations that have the best model fit, are reported across multiple large cohorts, and/or have undergone sensitivity analysis via VoE."

"However, if -- in part by modeling VoE -- we are able to identify robust-to-model..."

It does not explore nor compare with other methods, how much of these associations will end in being false positive / false negative.

We agree this is a drawback of our study (especially with regard to the previous point regarding the conditions needed for a robust discovery), and we now explicitly write in the Discussion:

"It complements existing approaches, such as Bayesian Model Averaging[32], whose primary goal is to provide an optimal single predictor by averaging across the many different models. Therefore, we posit here that VoE, which in future work should be compared to these other methods, should at present be used primarily as a way to probe associations from different modeling strategies to systematically assess the combination of potential adjustments."

"Furthermore, an ostensibly robust association viewed only through the lens of VoE still could be a false positive."

At the very least the discussion section should be toned done, and further developments necessary to develop and adopt this new approach must be suggested;

We have aimed to tone back our initially overzealous discussion into something more measured that both captures the core advance of the paper and also discusses what further developments are needed for robust discoveries in addition to robust associations. We specifically have:

1) Restructured the first paragraph to focus on the results of the paper
2) Explicitly stated that we did not pre-register and the drawbacks therein
3) Added a statement describing that we did not explicitly compare to other techniques
4) Clarifying that a robust association from VoE is not sufficient on its own for a translatable finding, making it one (not infallible) component of building a theoretical association prioritization framework
5) Clarifying that we do not explicitly build an association prioritization framework in this study

I leave the editor judge whether these concerns rather qualify for a major revision or for a rejection. I may suggest major revision but I think that an in depth revision of the discussion will be needed.

We thank the Reviewer and the Editors for the opportunity to revise our text, and we hope our heavy modifications to the Discussion are now satisfactory.

The paper is clearly written, understandable for a large audience despite many technical aspects. I do think that authors did a good job in this regard. Of course it could be improved and I hope that the following minor comments will be helpful.

1.3) COI disclosure: I would be interested to know more about the 2 companies (FitBiomics) and Micro Bioscience, and especially how links with these companies may represent a conflicting interest.

We have now updated our COI to be more specific. Micro Biosciences is a company with an employee of 1 (Dr. Tierney) that he founded after his PhD with the intent to use it for consulting as-needed. Given that the latter is neither relevant to this paper nor producing any products, we now simply have adjusted the COI statement to mention the 1 company he is working with as well as the nature of that work. We additionally have clarified Dr. Kostic's role in FitBiomics. We write:

"A.D.K. is a co-founder of FitBiomics, Inc. and a member of their Scientific Advisory Board. B.T.T. consults for Seed Health on microbiome study design and analysis."

1.4) Authors must give a close attention to their figures :

. In one hand figures are very nice to read and nicely summarize many very important information ;
. In the other hand, figures are very difficult to read (for each figure, it took me some times to understand the key concepts as these figures are not so intuitive) ;
. For example, in Figure 1, information about the search strategy is somewhat hard to understand, especially if you did not have looked at the method section. Figure 1 could be more aligned with the data reported in the text to make its reading easier.
A/ I appreciate that y axis are in a log scale / however, the figure should provide p-values (on a log scale) rather than log p-values. It would be easier to read. This could apply to all figures.
B/ In Figure 1 A, there is no information of the y and y axis for the VoE, taxon graphs. I think that it could be difficult to read.
C/ In figure 1B and 1G, there is no label for the x-axis. The number 1 to 6 could be difficult to follow. I would suggest to think in more user friendly representation.

In accordance with these suggestions, we have made the following changes:

F1A -- We have focused the text away from the literature review aspect and more on the overall associations/recovery after VoE. As a result, we now show in F1A that we are extracting microbial data and comparing a subset with associations from the literature, reordering the flow of the diagram for clarity. We have added axes where requested.

F1C-F -- These are now figures F1B-E (so that formerly F1B/G are now side by side and more easily compared. Also as requested, have now plotted raw p values on a log scale (so the y-axis is now decreasing instead of increasing but is more interpretable).

F1B/G -- These figures are now combined and separated out into a new Figure 2. We have removed the 1-6 annotation on the x axis and colored bars to clarify our overall point.

We additionally have replaced log values with equivalent raw p-values in all figures in the manuscript.

We reproduce the Figures in question (now 1 and 2) and its associated legend here:

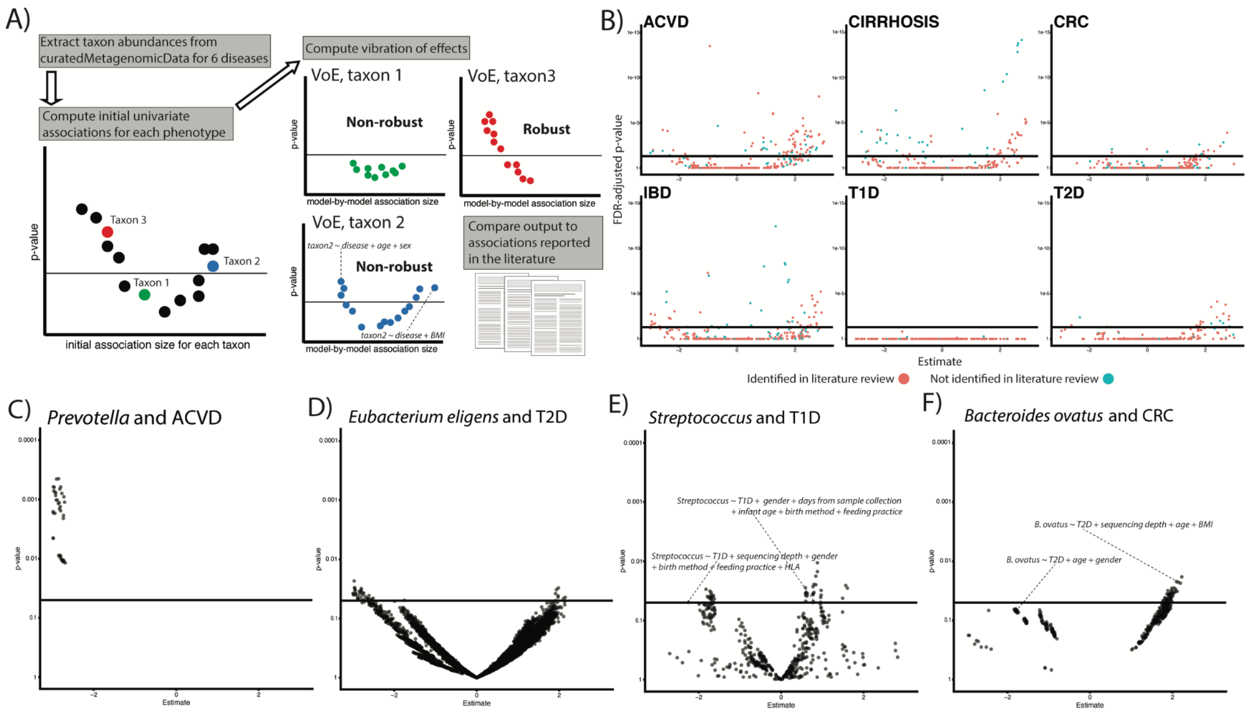

Figure 1: A) Overview of approach. We extract prevalent microbial features from our datasets and attempt to reproduce the findings from the literature by modeling vibration of effects. We additionally review the literature for reported gut microbiome associations (their reported direction of correlation) with six diseases of interest. B) Volcano plots showing the output from the initial, univariate associations. Point color corresponds to if an association was identified in our literature review solid line represents FDR significance (adjusted $p < 0.05$). C-F) Examples of robust (C) and non-robust associations. Each point represents a different modeling strategy. Solid line is nominal ($p < 0.05$) significance.

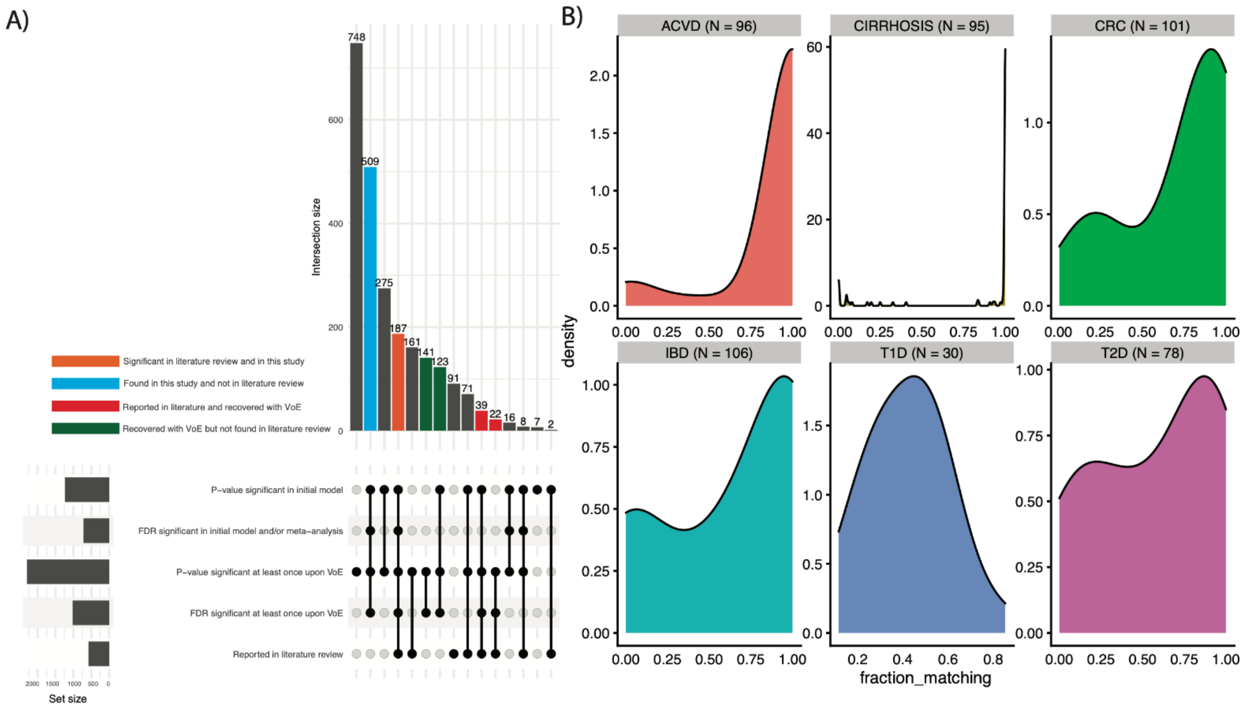

Figure 2: Comparing single modeling approaches to modeling VoE. A) Stratifying features by their being identified in our literature review, p-value/FDR significant (< 0.05) in our initial analysis, or having at least 1 significant model upon vibrations. B) Distribution of fraction of models matching literature-review-defined direction of associations.

. For example, in Figure 2 :
A/ The Figure use the term Janus Effect in its legend. It is of course defined in the figure but it does not appear in the text. I would suggest that authors could add this in the text;
B/ There is no information about the axes (no label for the y nor for the x axes). It could be difficult to follow. I don't have any good suggestion for improving the readility and adding more information about the associations being studied / but I suggest that authors may think in a more user friendly figure ;

To make this paper more friendly to microbiome scientists, we opted to not explicitly use the phrase “Janus Effect,” however these instances slipped past us. We thank the Reviewer for this catch and have updated accordingly. We additionally have attempted this figure to be more readable by adding y and x legends.

. For example, in figure 3A, I would use the same scale for all effect size across all diseases (e.g. using a log scale) ;

We understand that the arrangement of this figure (e.g. through a shared y axis) made it appear that axes were meant to be compared. Given the different cohorts, phenotypes, and variables, we in fact want readers to consider each plot separately, and putting them on the same scale, we think, would potentially obscure some of the interesting, smaller association sizes. To

address this point, we have split panel A into 6 sub-panels to indicate our intent for them to be analyzed separately.

1.4) P.4 l.98 37.8 findings were from the datasets used in this analysis. I would be interested to know more about reproducibility of a finding in the dataset where this finding is from versus in other datasets (e.g. in line 109-110, I would be interested to break this by original versus new datasets). It could be out of scope but I would like to hear the authors'point of view about this point.

We agree this is an interesting point. While we feel a deep dive on it is outside the scope of this manuscript, especially given that doing so fully (to get at reproducibility) would likely require emulating the modeling strategies used in the studies in question (for more on why we are avoiding claiming to be measuring reproducibility, please see points 2.1 and 2.8).

However, to address this point, we do now report the number of features that were FDR-significant at least as a function of if they were reported in the studies in our database or otherwise. We write in the Results:

“This brought the total number of taxa-disease associations we were able to recover (i.e. achieve FDR significance at least once) to 248. Of these, 117 were not reported in the datasets used in this study and 131 were. ”

1.5) P.4. l.111, I'm not sure that the title is adequate. Instead of "additional potential", I would suggest "different". In this paragraph I would also be interested in Venn's diagram showing the overlap between usual approach and the approach proposed by the authors ;

First, we have made this phrasing change as requested.

Second, we hesitate to explicitly generate a Venn Diagram comparing our results to a “usual” method (outside of what is now shown in Figure 2), however, in part due to some comments raised by the second Reviewer(s) (2.8, among others, which we agree with). Microbiome analyses are so wildly non-standardized that there really is no usual approach, and fitting a univariate association is likely not to be representative of the cutting edge across the discipline. Indeed, the cutting edge varies on a study by study basis and in the eye of the statistician in question (which brings us back to our motivation for fitting as many models as possible through VoE).

We aimed to highlight here the difference between fitting 1 model (which is typical, most studies don’t try more than one) vs. fitting as many as possible. It is for this reason we hesitate to even use the word reproducibility or even state that we are explicitly testing results from the literature instead of expanding on them. Since everyone is using different approaches, testing their results would require recreating the approach, which is such a grand project we feel it is outside the scope of this VoE use-case.

1.6) p5. l.119 : "we hypothesized" OK : was a it a priori ? Or was it a posteriori (see my previous comment) in major concerns ;

This was not an *a priori* hypothesis, and we have removed the reference to "hypothesizing" here. Instead, we state what happened, which is that VoE did yield some new, potentially interesting associations:

"...modeling VoE was able to both shed light on associations that would be potentially overlooked by single modeling strategies, in some cases recovering results reported in the literature."

1.7) p.6. l.159-161. This is rather an interpretation and should be moved in the discussion section. And, also, following my second major concern, authors must provide convincing evidence to support such a statement. I'm not sure that it is possible based on their data.

We have moved this statement to the Discussion, as we agree it is interpretation.

We also have clarified the phrasing to indicate that in the case of a single p-value cutoff, they may miss potentially interesting biology that happens to fall outside of significance simply due to model specification. This is yet another case study (like in point 1.8) that we decided to include as an example after our analysis was complete. In line with our "discovery-based" approach, we could not know beforehand if we would find *Roseburia* (or any taxon) to have many models (but not the baseline) to be statistically significant. We happened to notice that this was the case, though, and decided it was worth having in the manuscript to indicate how VoE can reveal these potentially interesting associations that may be overlooked.

1.8) p7. l193: Here again I'm surprised that the author selected a specific example (i.e. F. Prausnitizii). Was this done a priori. Why this specific association? I would have preferred using specific criteria and a random selection of example using the predefined criteria. I may have missed something however.

We thank the reviewer for pointing this out, as we can see how it might seem unjustified to select this particular organism. We have clarified in the text this decision, and we will expound here as well.

We chose to look at *F. prausnitzii* predominantly because of its relevance to the microbiome field, as gauged by its large presence in our literature review. Upon surveying the results of the literature review, our team noticed *F. prausnitzii* was associated with multiple phenotypes. Indeed, this organism is of immense interest to the field -- it is difficult to find a single microbiome association study that fails to mention it. Here are a number of studies that do (we now cite them in the Results):

https://www.sciencedirect.com/science/article/pii/S1521691817301063
https://link.springer.com/article/10.1186/1752-0509-8-41

https://academic.oup.com/ibdjournal/article/15/8/1183/4643499?login=true
https://www.ingentaconnect.com/content/ben/emiddt/2016/00000016/00000002/art00006
https://www.sciencedirect.com/science/article/pii/S1369527413000775

As a result, upon examining both the literature review and the results of our VoE analysis (which found *F. prausnitzii* to be significantly associated with many diseases), we chose to hone in on it as a case study, as we felt ending the paper with a specific example would be useful to readers to understand how VoE can be used to look at a single organism. We do not think it would have been feasible to claim from the outset that we planned on looking at this microbe, as we did not know it would be found in the literature review or found to be robustly associated with so many phenotypes. So, in summary, given its importance to the field and prevalence in our dataset/results, we opted after the fact to consider *F. prausnitzii* specifically in our manuscript.

We are aware, though, that this discovery-based approach certainly is lacking in many dimensions. We mention this in the discussion now (see point 1.1), and we also point out the potential downside of not preregistering. We also write:

“This is particularly difficult for hypothesis-generating studies, though, as the outcomes cannot be preset -- for example, our decision to focus on *F. prausnitzii* as a case study could not have been made until seeing the results.”

1.9) P.8 l.206: I think that the very first sentence of the discussion must summarize the findings. I would therefore suggest to delete (or move in the introduction) the very first sentences.

We agree this was an inappropriate way to begin the Discussion, and we have rephrased this first paragraph to focus initially on our results. We write:

“In this study, we explored the utility of modeling Vibration of Effects for Microbiome-Association-Studies. Across a number of diseases with varying number of cohorts, sample sizes, and metadata, we showed how massive-scale, automated sensitivity analysis can be used to 1) query how associations found in the literature may change as a function of model choice, 2) identify overlooked associations (such as the association between *Roseburia* and ACVD, which a single modeling approach may miss) identify sets of potentially important, disease-specific confounding variables that should be accounted for in current and future MAS.”

1.10) P. 10 l.261-263: In line with my previous comment, I would be interested to know more about discrepancies between re-analysis in a given dataset and the original analysis.

We agree this is very interesting -- please see point 1.4.

1.11) p.14, l.422: How the 20 adjusting variables were selected. I think that this point could benefit from more details.

Thank you for pointing this out. The correct number and type of adjusting variables is certainly something that needs to be addressed in future work (and we hope it can be done with our package). We chose 20 here simply because it guaranteed a sufficiently large sampling of variable combinations. However, motivated by this comment and others, we have carried out an analysis where we modify both the total number of models and adjusting variables in a given model for Type 2 Diabetes to demonstrate that there is limited effect on ostensible robustness as a function of these parameters and the variable types. You can see the output of this analysis here, as well as in a new Supplemental Text section, where we write:

“We additionally benchmarked additional aspects of our VoE approach for our T2D cohorts, which contained the largest number of adjusting variables in our dataset. We compared different data transformations prior to modeling (centered-log-ratio [CLR] vs. log-transformed vs. unmodified abundance values) as well as vibrations with different numbers of variables. Different transformation methods yielded different results: log-transforming variables resulted in the largest number of associations that were at least FDR significant once (Supp Fig 1A-C). The number of vibration variables also slightly changed output, with increasing number of adjusters (and therefore vibrations) yielding more taxa, as one would expect that were FDR-significant at least once, indicating a potential drawback of misuse of VoE. That said, between vibrating over 3, 6, and 9 variables, the number of potentially significant features for meta-analyzed, log-transformed datasets went from 19 to 30 to 31, indicating a potential leveling-off with increasing adjusting variables.

Finally, we estimated changes in ostensible robustness of different associations for the three data transformation strategies and number of variables vibrated over (Supp Fig 1D). For all taxa, estimated the correlation between the fraction of all associations for a given taxon that were positive (a measure of how consistent, or robust, an association is). Highly robust associations have entirely positive (fraction approaching 1) or entirely negative (fraction approaching 0) association signs. Non-robust associations have fractions closer to 0.5 (i.e. 50% of models producing conflicting results. We found high correlations (>.9) between these values for given transformation methods regardless of the number of vibrations selected. In other words, a robust association was consistent regardless of the number of vibration variables selected.”

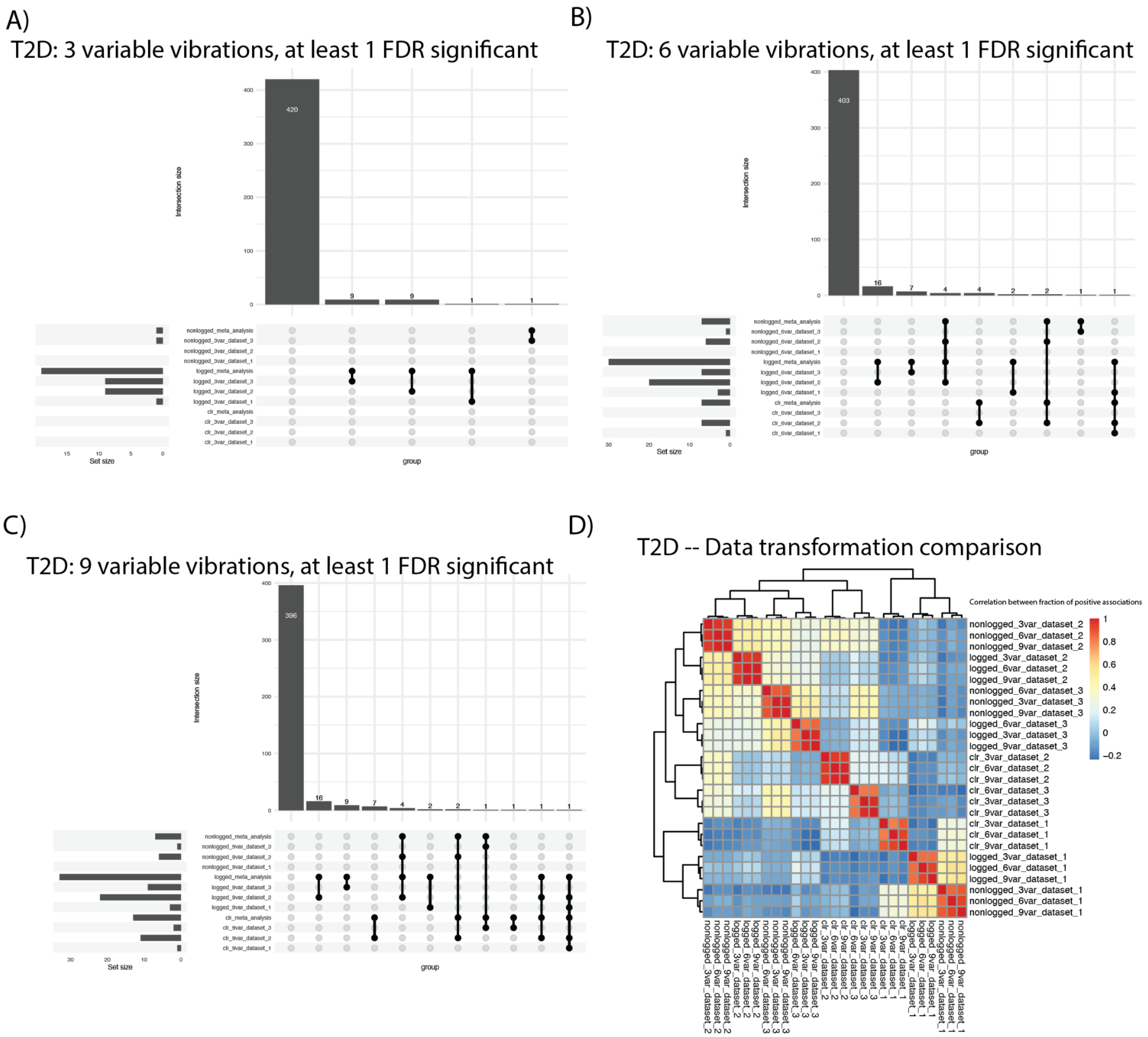

Supplementary Figure 1: Benchmarking data transformations and the number of adjusting variables using our T2D datasets. A-C) The impact of the different numbers of vibrations and data transformation methods on vibration of effects. We plot the number of features that were FDR-significant at least once upon vibration with different numbers of adjusting variables considered as well as different data transformation strategies (i.e. logged vs raw abundances vs center log-ratio transformations). D) The robustness of associations as a function of number of vibration variables and modeling strategy. We computed the fraction of associations that were positive for any given microbial feature -- a highly robust association is 100% positive or 0% positive (i.e. negative), whereas a non-robust association is closer to 50% positive (i.e. inconsistent in direction). In this heatmap, we correlated these associations for all features to gauge if the different data transformations and numbers of adjusting variables yielded similar measures of robustness across all datasets.

Later in the results, we additionally reference another benchmarking figure regarding the number of variables selected and vibrations performed:

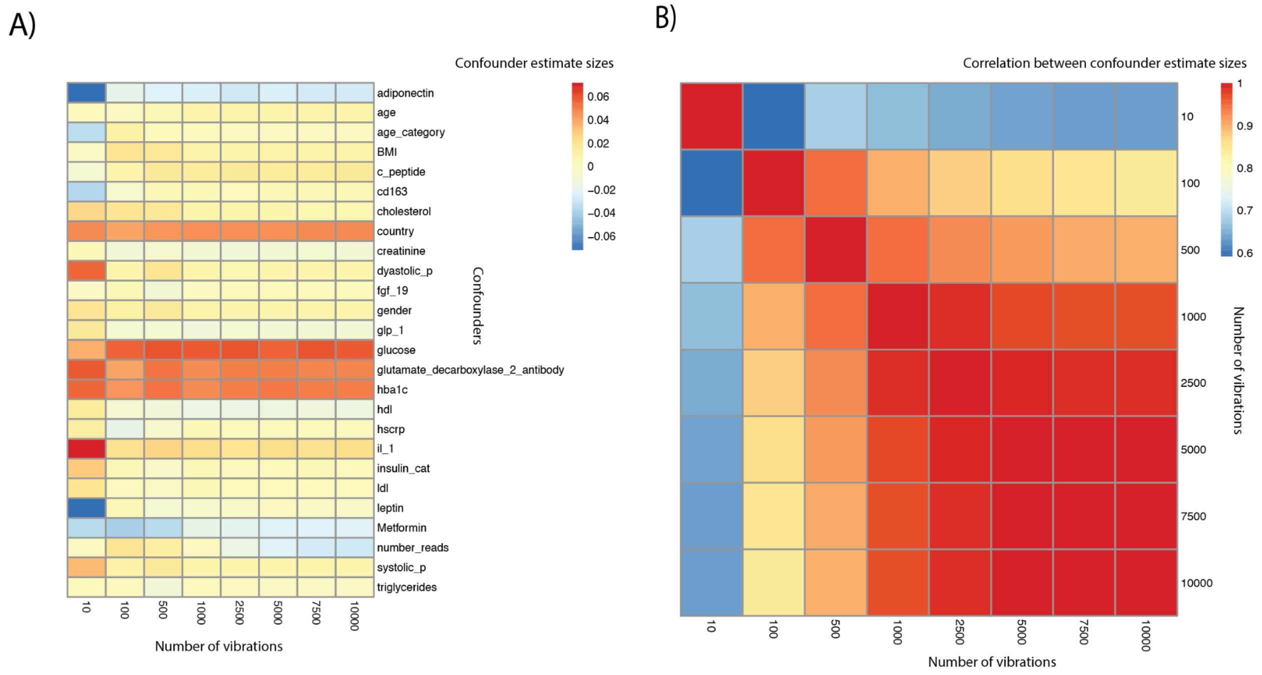

Supplementary Figure 4: Benchmarking the number of vibrations needed to estimate the effect of confounding on microbiome associations. A) The output of our confounder analysis (e.g. in Fig 4). The x-axis is the number of vibrations. The y-axis is each possible adjusting variable in the T2D associations. The values correspond to the beta-coefficient (from our mixed effects analysis) describing the average change in microbiome-disease associations when a given adjusting variable is present in a model. B) The correlation between the values in panel A at different numbers of vibrations.

Regarding this Figure, we write:

“As a form of benchmarking, we estimated in the T2D cohort how the beta coefficients on the adjusting variables from the mixed modeling approach changed as a function of the number of vibrations executed (Supp Fig 4). We found 10,0000 vibrations (our upper limit) to be sufficient to identify consistent correlation between these beta-coefficients (pearson >.9).”

Reviewer #2

This paper explores the microbiome field's need to account for the portion of bias introduced by confounding variables when choosing models of association between bacterial taxa and human disease. The authors chose to address this through a process called "Vibration of Effects." In addition to identifying associations that change when adjusting for potential confounding variables, the authors promise to:

Measure reproducibility of previous literature findings of association,

Identify new biological associations between bacterial taxa and human disease,
Provide recommendations on feature prioritization in future studies, based on their ability to recapitulate associations in this work.
Identify whether an adjuster is of the confounder or collider variety.

We appreciate this summary, as taken with the proceeding comments (as well as Reviewer 1's comment regarding the need to walk back some claims), they laid clear what we needed to revise in terms of the goal of our manuscript. While we touch on a subset of points, we do not mean to claim to achieve completely everything in this list. To set expectations up front, we now write in the Introduction:

"Here, to gauge the impact of model specification in MAS, we deploy a systematic sensitivity analysis, measuring Vibration of Effects in reported microbiome associations. Comparing modeling strategies, we quantify the robustness(variation as a function of model specification) in microbial taxon-disease associations across six different phenotypes. With an emphasis on 581 associations that were reported in the literature, we counted how many associations (published and otherwise) are recovered (i.e. appear as statistically significant) when undergoing sensitivity analysis. We propose modeling VoE as one of many potential steps in building association prioritization frameworks, metrics for prioritizing microbiome findings for *in vivo* validation."

This change is in addition to a number that are described in response to the following points.

Major comments:

2.1) The authors state that VoE can be used to assess reproducibility, but the most non-robust associations were also those that had the most available covariates, and therefore yielded the highest number of models. This process seems highly dependent on the type and number of adjusters available in sequencing metadata. VoE seems better suited to evaluating associations after a study without attempting to narrow in on a one, best model.

We agree with the sentiment of this point -- VoE is certainly dependent on the number of and type of available covariates (and therefore possible models). Further, its optimal application is certainly not to find a "best" model -- rather, as the Reviewer states, it is better suited to evaluating associations after a study.

We now describe this drawback explicitly in the Discussion:

"...as we observed in **Supp Fig 1**, vibration may be contingent upon the number and type of variables measured, the size of the cohort, and variability of the measurements."

However, in addition to mentioning this, to fully address the Reviewer's comment we, for T2D (for which we had the largest number of variables and thereby could test the largest number of vibrations), carried out a variable selection sampling analysis where (when possible). We selected either 3, 6, or 9 variables at random and ran our VoE pipeline to compute overall

associations and VoE. We found consistent VoE regardless of the number of variables sampled (in Supp Fig 2D and Supp Fig 4, reproduced below) as recently documented in our companion manuscript (https://journals.plos.org/plosbiology/article/metrics?id=10.1371/journal.pbio.3001398).

While of course this does not abnegate the Reviewer's point, we hope that it provides some confidence that non-robust associations, at least in this paper, are likely non-robust regardless of the number and type of variables sampled and measured in the observational cohort, respectively. Naturally, though, in observational, publicly available, data we are at the mercy of those who collected it initially, and there are almost always certainly more informative variables to measure than what is available.

We reference these results in the following Figures, which are reproduced here

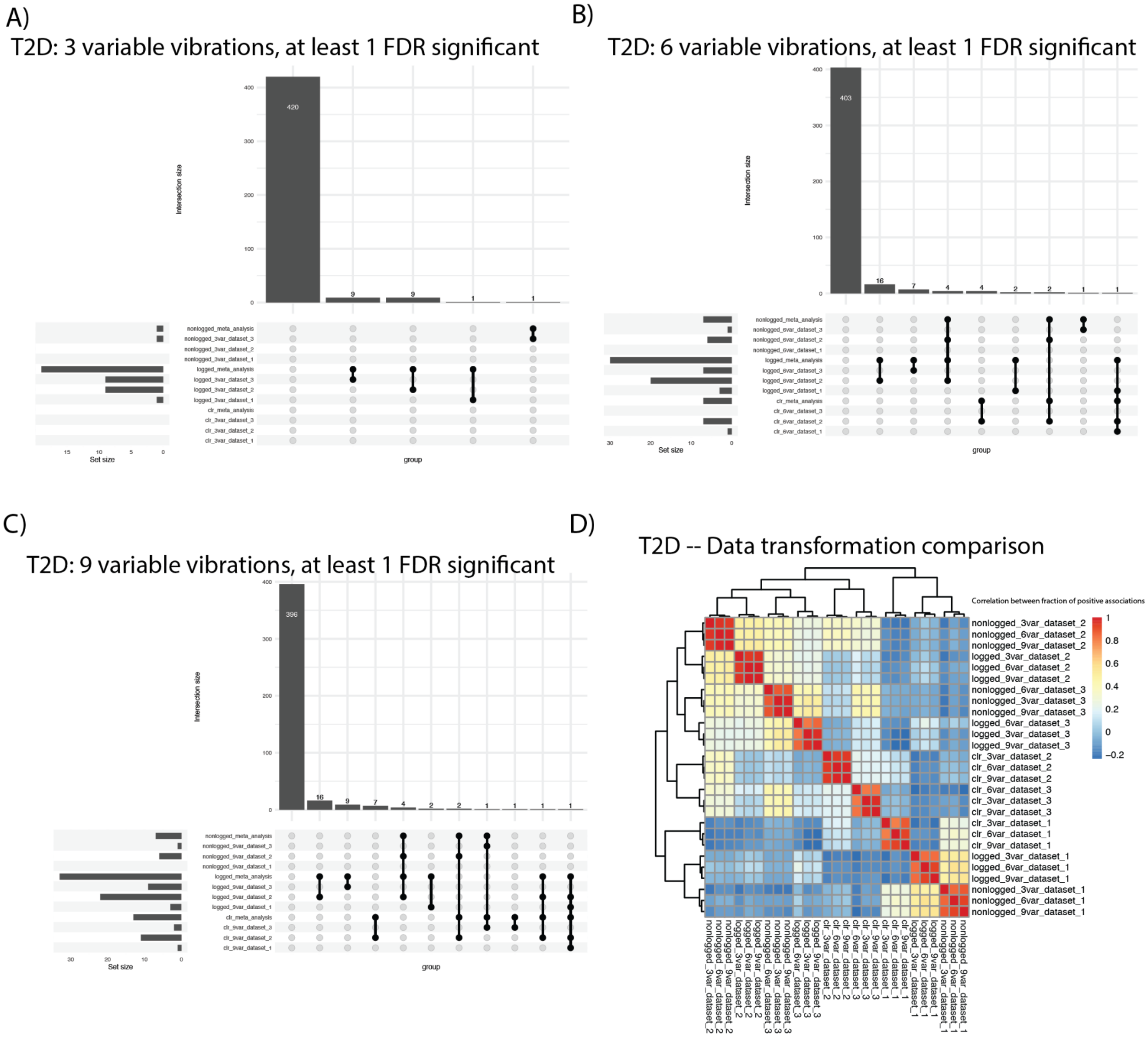

Supplementary Figure 1: Benchmarking data transformations and the number of adjusting variables using our T2D datasets. A-C) The impact of the different numbers of vibrations and data transformation methods on vibration of effects. We plot the number of features that were FDR-significant at least once upon vibration with different numbers of adjusting variables considered as well as different data transformation strategies (i.e. logged vs raw abundances vs center log-ratio transformations). D) The robustness of associations as a function of number of vibration variables and modeling strategy. We computed the fraction of associations that were positive for any given microbial feature -- a highly robust association is 100% positive or 0% positive (i.e. negative), whereas a non-robust association is closer to 50% positive (i.e. inconsistent in direction). In this heatmap, we correlated these associations for all features to gauge if the different data transformations and numbers of adjusting variables yielded similar measures of robustness across all datasets.

We reference this Figure by writing (in Supplemental Text):

“We additionally benchmarked additional aspects of our VoE approach for our T2D cohorts, which contained the largest number of adjusting variables in our dataset. We compared different data transformations prior to modeling (centered-log-ratio [CLR] vs. log-transformed vs. unmodified abundance values) as well as vibrations with different numbers of variables. Different transformation methods yielded different results: log-transforming variables resulted in the largest number of associations that were at least FDR significant once (Supp Fig 1A-C). The number of vibration variables also slightly changed output, with increasing number of adjusters (and therefore vibrations) yielding more taxa, as one would expect that were FDR-significant at least once, indicating a potential drawback of misuse of VoE. That said, between vibrating over 3, 6, and 9 variables, the number of potentially significant features for meta-analyzed, log-transformed datasets went from 19 to 30 to 31, indicating a potential leveling-off with increasing adjusting variables.

Finally, we estimated changes in ostensible robustness of different associations for the three data transformation strategies and number of variables vibrated over (Supp Fig 1D). For all taxa, estimated the correlation between the fraction of all associations for a given taxon that were positive (a measure of how consistent, or robust, an association is). Highly robust associations have entirely positive (fraction approaching 1) or entirely negative (fraction approaching 0) association signs. Non-robust associations have fractions closer to 0.5 (i.e. 50% of models producing conflicting results. We found high correlations (>.9) between these values for given transformation methods regardless of the number of vibrations selected. In other words, a robust association consistent regardless of the number of vibration variables selected.”

Later in the Results, we additionally reference another benchmarking figure regarding the number of variables selected and vibrations performed:

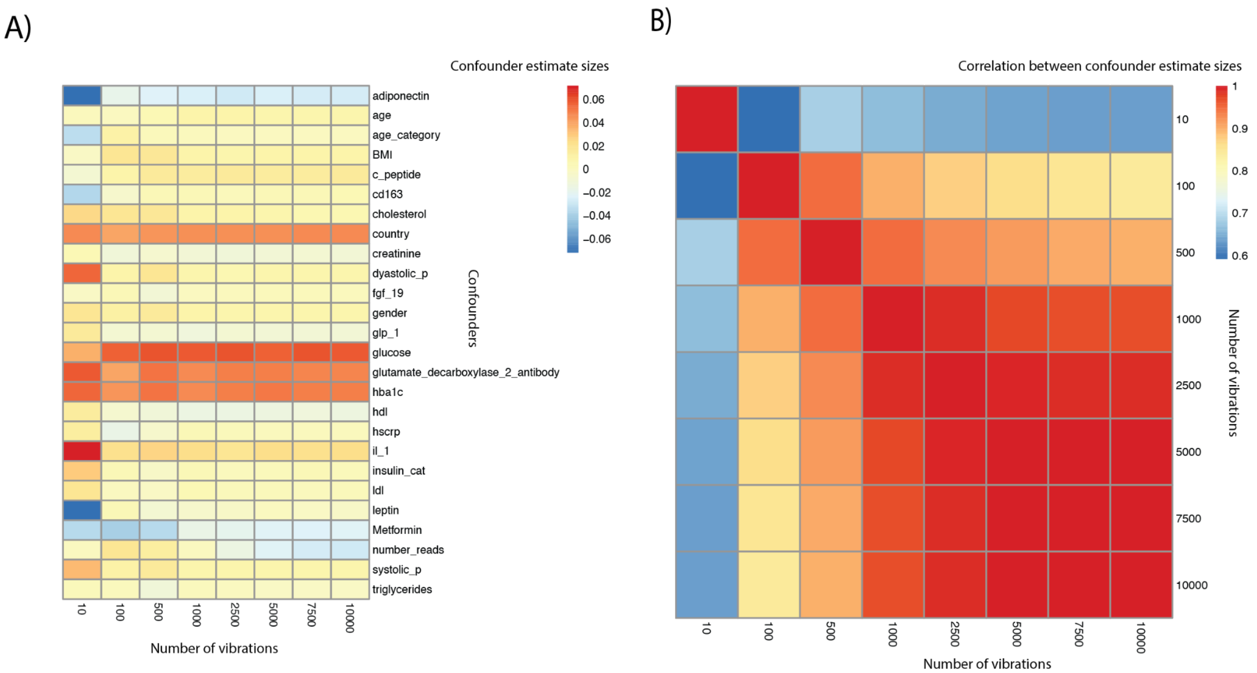

Supplementary Figure 4: Benchmarking the number of vibrations needed to estimate the effect of confounding on microbiome associations. A) The output of our confounder analysis (e.g. in Fig 4). The x-axis is the number of vibrations. The y-axis is each possible adjusting variable in the T2D associations. The values correspond to the beta-coefficient (from our mixed effects analysis) describing the average change in microbiome-disease associations when a given adjusting variable is present in a model. B) The correlation between the values in panel A at different numbers of vibrations.

Regarding this Figure, we write:

"As a form of benchmarking, we estimated in the T2D cohort how the beta coefficients on the adjusting variables from the mixed modeling approach changed as a function of the number of vibrations executed (Supp Fig 4). In other words, we computed how the estimated impact of an adjusting variable being present in a model changed as a function of number of vibrations completed. We found 10,0000 vibrations (our upper limit) to be sufficient to identify consistent correlation between these beta-coefficients (pearson >.9)."

We also describe this approach in the Methods:

"*Benchmarking data transformations, adjusting variables, and vibration numbers*
We used the T2D datasets (which have, in total, the with the most number of potential adjusting variables recorded) to compare the impact of different data transformations, adjusting variables, and vibration numbers on our results. We compared center-logged-ratio (CLR) transformations on each dataset, our logging strategy described above, and the raw abundance data, running our entire pipeline with 10,0000 vibrations. We additionally compared our results when 3, 6, or 9 variables were selected at random from each cohort.

As a measure of robustness, we computed the fraction of all associations that were positive, with fractions approaching 1 or 0 being highly robust, and fractions approaching 0.5 being non-robust (e.g. reporting conflicting results close to half of the time). For Supp Fig 1D, we computed the Pearson correlation between these fractions.

Finally, we additionally compared the results of the mixed effects confounder analysis as a function of number of vibrations fit. In Supp Fig 4, we report the output of this analysis and the correlation between the estimate impact of each adjusting variable on model output."

On a broader note, we are particularly grateful for this comment, as it illuminated an interpretation we wish to avoid with our manuscript -- the focus on reproducibility. We wish to clarify that we do not mean to claim that VoE is a way of measuring the reproducibility of associations explicitly. Doing so, we believe, would require 1) a systematic review and 2) using the exact modeling strategies and study designs as reported in the manuscripts in question as the baseline model. Indeed, as the Reviewer points out in future comments (points 2.8, 2.12, 2.15), comparing a "baseline" (unadjusted) model to all others may be unfair if the baseline model is not the one fit in the initial manuscript.

In the submitted draft, we mentioned briefly mentioned this in the Discussion, describing how the word reproducibility is so loaded (and negative) that it almost isn't worth explicitly discussing:

"We do not claim our approach is the be-all-and-end-all of microbiome statistical sensitivity analysis. It is merely one metric for quantifying association robustness. It is for this reason that we do not claim that we are holistically measuring reproducibility, as the requirements for rigorously doing so are nebulous[23] and would likely require considering all biases that pervade microbiome analyses, something outside the scope of this project."

For this reason (as Reviewer 1 identified), we attempted to take a different approach, discussing the discovery of confounders and the ability to identify potential associations of interest that would be missed by a single modeling strategy. That said, we clearly did not communicate this point effectively in the previous version. After some consideration, we believe we identified why our writing failed to effectively communicate our intended purpose and instead erroneously gave the impression we were testing reproducibility (for example, the Github repository quite literally had the word reproducibility in its title, and we mention the concept in the first paragraph of the introduction). We have now updated the manuscript to clarify our aims (and we also renamed the Github repository).

For example, we now open the introduction to write:

Here, to gauge the impact of model specification in MAS, we deploy a systematic sensitivity analysis, measuring Vibration of Effects in reported microbiome associations. Comparing modeling strategies, we quantify the robustness(variation as a function of model specification) in microbial taxon-disease associations across six different phenotypes. With an emphasis on 581 associations that were reported in the literature, we counted how many associations (published

and otherwise) are recovered (e.g. appear as statistically significant) when undergoing sensitivity analysis. We propose modeling VoE as one of many potential steps in building association prioritization frameworks, metrics for prioritizing microbiome findings for *in vivo* validation."

2.2) In addition to the number of measured covariates, VoE appears to be sensitive to the number of cohorts, sample size, and variability in measurements. These differ across the studied diseases. When comparing results over diseases (Lines 140-145, for example) these factors should be discussed even more explicitly. Single-cohort associations are mentioned, but it is not clear if cohort is one of the covariates included in VoE or could be included. Providing simulations or down-sampling experiments on the larger data sets to show how these variables affect VoE in this application would be extremely useful.

We agree that this point is important and should have been clearer in the initial manuscript. As a result, we have now aimed to clarify that observed VoE will be contingent on the greater study design, including the type of data (e.g., species vs. metagenomic data), the number of cohorts being combined, and their individual sample sizes.

First, to immediately address this concern, as we mentioned above, we now mention in the discussion that VoE is sensitive to the above described features:

"as we observed in Supp Figs 1 and 4, vibration may be contingent upon the number and type of variables measured, the size of the cohort, and variability of the measurements."

To further clarify this point here: we had data on three diseases (T2D, T1D, CRC) with multiple cohorts, whereas the other three diseases only had one cohort. The tool we used to analyze these datasets, quantvoe (the subject of a project that came out [Tierney et al., *PLOS Biology 2021*] while we were working on these revisions), automatically meta-analyzes (i.e. combines associations) data from multiple cohorts. This is how we got to final summary statistics for each phenotype -- we now clarify this in the introduction:

"Three of these diseases (T2D, T1D, and CRC) had participant data across multiple cohorts. For these, we meta-analyzed across individual associations within each cohort to compute overall (cross-cohort) summary statistics or associations."

Further, to illustrate this point, as recommended, in Supp Fig 1 (reproduced above in point 2.1), we detail the similar output between individual cohorts (and different data transformation strategies).

We show, indeed, that results vary across cohorts, and we compare how the meta-analyzed results differ from the individual cohort results. We also showed (as referenced in point 2.1 with regards to Supplementary Figure 4), however, that the number of variables measured had limited impact on the robustness of associations. In other words, a non-robust association was consistently non-robust regardless of the number of variables measured.

2.3) The premise for using VoE needs further justification. Why would a marginal association be inherently better if it differs little from all possible conditional associations? Identifying confounding variables makes sense, but it seems like this method is broader than that. Justification needs to be provided for seeking reproducibility over models with all possible/measured variables given that the authors acknowledge that some covariates should not be adjusted for. Similarly, why is a microbe-disease association inherently more interesting if it is reproduced across multiple diseases (Lines 226-228 for example)?

We agree that there were many assumptions implicit in our writing that could have been more explicit. We have sought to update the Introduction and Discussion to address these questions of motivation. We additionally cite more manuscripts that outline the use and justification for Voe.

In the Introduction, we now write:

“These analyses may be particularly useful for discovery-based studies (very common in the microbiome and genomic fields), approaches designed to generate, rather than test specific candidate, hypotheses from complex datasets.”

“Here, to gauge the impact of model specification in MAS (with reported inconsistency in microbiome association as as the justification for doing so), we deploy a systematic sensitivity analysis, measuring Vibration of Effects in reported microbiome associations”

“We propose modeling VoE as one of many potential steps in building association prioritization frameworks, metrics for prioritizing microbiome findings for *in vivo* validation.”

And in the Discussion:

“We claim that one step -- out of many -- towards translating microbiome findings into biological understanding is determining how best to prioritize for future (e.g. *in vivo*) investigation associations arising from MAS.”

“That said, modeling VoE is certainly not the only way to identify an association worth prioritizing. Furthermore, an ostensibly robust association viewed only through the lens of VoE still could be a false positive or dependent on, for example, data processing pipeline choice (e.g. the decision to average repeated measures data vs. selecting one sample per individual). A more comprehensive framework could rely on a number of heuristics, for example putting the greatest emphasis on associations that have the best model fit, are reported across multiple large cohorts, and/or have undergone sensitivity analysis via VoE.”

We additionally now reference a manuscript detailing and justifying VoE as a method (and presenting the package used in this analysis) that has been published in the intervening period between receiving and responding to reviews on this paper:

https://journals.plos.org/plosbiology/article/peerReview?id=10.1371/journal.pbio.3001398

Other manuscripts, also referenced in this one, that justify VoE as a method (e.g. even when colliders, for example, are present) are listed here:

https://www.sciencedirect.com/science/article/pii/S0895435615002772
https://academic.oup.com/ije/article/49/2/608/5714100?login=true
https://bmcmedicine.biomedcentral.com/articles/10.1186/s12916-019-1409-3

Regarding why microbes associated with multiple diseases are interesting -- our writing this line was perhaps a latent bias that is in contrast to established heuristics for gauging associations (e.g. Bradford-Hill -- https://www.ncbi.nlm.nih.gov/pmc/articles/PMC1898525/). We hypothesize that microbes associated with multiple diseases may potentially be useful for diagnostics and/or potentially play a role in multiple disease etiologies through a conserved gut microbial mechanism. To address the reviewers comment, though, we removed the phrase "and diseases" from the text.

2.4) An association-prioritization framework of variable adjusters that must be considered across multiple diseases and studies is promised as a result, but not provided by the manuscript.

We thank the Reviewer for this comment, as it highlights what we view to be a very important point that was clearly not communicated effectively enough.

First, to clarify, we define an association-prioritization framework not as a set of adjusting variables that must be considered across multiple diseases, but rather ranking associations (e.g. microbe X and disease Y) by priority for follow up (e.g.experimental or wet laboratory) investigation. The sheer volume of associations reported in microbiome studies cannot, at present, all be tested *in vivo*, so we need ways for picking which ones are worthwhile. We propose modeling VoE as one potential dimension of this decision making (out of many options). We additionally believe that associations reproduced in multiple studies (via a meta-analysis, as we do for some cohorts), as well as those that are consistently associated with disease (the point we make in 2.3), are worth strongly considering. As Reviewer 1 points out (in point 1.3), such a framework that guarantees translatable associations is likely not possible from VoE alone.

To get at this point, we initially wrote:

"We need "association-prioritization frameworks:" the contexts in which of these manifold associations are most worthy of wet lab vs. computational follow up and which are not worth

pursuing that complement existing approaches to improve, or omit, analytic robustness.[3,27,28].”

However, this line appeared in the Discussion, and we did not effectively define association-prioritization frameworks when they are first introduced, in the Introduction. We now do so, and we clarify our intent in demonstrating how VoE can slot into the generation of such a framework:

“We propose modeling VoE as one of many potential steps in building association prioritization frameworks, metrics for prioritizing microbiome findings for *in vivo* validation”

Additionally, in the process of reworking the Discussion, we have updated our discussion of this topic, writing:

“We claim that one step -- out of many -- towards translating microbiome findings into biological understanding is determining how best to prioritize for future (e.g. *in vivo*) investigation associations arising from MAS. There is a need for association-prioritization frameworks: the contexts in which of these manifold associations are most worthy of wet lab vs. computational follow up and which are not worth pursuing that complement existing approaches to improve, or omit, analytic robustness.[3,30,31]. In this study, we do so by identifying which associations are consistent in direction and statistically significant across multiple cohorts. We propose modeling VoE a single component of this...”

2.5) How are the findings and measurements of statistical significance affected by only analyzing previously significant microbe-disease associations (Lines 252-257)? Regression to the mean type thinking and extreme value statistics suggest that these will be biased towards non-significance and smaller effect sizes. It would be interesting to include non-significant results and look at how many become more significant. What is known about VoE conditional on starting with previously reported associations versus vomiting this conditioning?

We have now reworked our analysis to hopefully address this point -- to answer the last question, little is known about VoE condition on starting with previously reported associations.

As such, we no longer focus exclusively on the associations reported in the literature at the outset of the manuscript. Hopefully, this handles a number of issues brought up by the reviewers, including our literature review strategy, the focus on reproducibility (which we do not claim to be wholly testing), the use of a baseline, univariate, model, and of course, the issue referenced here: regression to the mean and bias in looking “under the lamppost,” so to speak.

We focus now on all microbial taxa in all cohorts and report those that are 1) initially significant as reported in the prior literature and 2) become significant after vibration. We additionally compare these analyses to the subset of features that we found to be reported in the literature, and discuss how to stratify associations into different categories:

“We next aimed to stratify associations by their 1) presence in the literature and 2) robustness/recovery as a function of vibration effects. A total of 509 features were FDR significant at baseline, additionally during VoE, and not reported in the literature review (**Fig 2A, blue column)**. 187 were FDR significant at baseline and reported in the literature (**Fig 2A, orange column**). An additional 61 of others (10% of all literature-based associations) found in the literature were FDR significant in at least one vibration but not in the baseline model (**Fig 2A, red columns**). This brought the total number of taxa-disease associations we were able to recover to 248. Additionally, 264 taxa were not found in the literature but were FDR significant at least once during a vibration (**Fig 2A, green columns**). In other words, we observed that modeling VoE was able to both shed light on associations that would be potentially overlooked by single modeling strategies, in some cases recovering results reported in the literature.

As another measure of robustness, for each taxon for each disease, we report the fraction of associations with signs matching the literature (**Fig 2B**). For all diseases except T1D, these distributions of the fraction of associations whose signs matched what was reported before was bimodal. The mode of the distribution was closer to 1, indicating a large frequency of high concordance associations and a moderate frequency of extremely low concordance (i.e. almost all models pointing the opposite direction as the literature) associations. Given the distribution of the data in Fig 2B, we defined a low concordance association as agreeing in direction in 50% of models fit. 27.9% of all features fit into this category; in other words, 1 in 3 features were discordant with the literature in 50% of all vibrations.”

Other comments:

2.7) Line 63: Explain VoE in more detail (text on Lines 419-422 seems helpful, for example) and any statistical theory justifying it as a metric for evaluating measured associations. Is there a causal inference framework underlying VoE?

We agree and have moved these lines to the introduction, where we write at the end of the third paragraph:

“In this manuscript, vibration of effects is computed by, for each microbial feature-disease pairing, fitting all possible linear models, each adjusted by different features, while tabulating how the association between the microbial feature and disease changes. Robust microbial feature-disease associations are those whose association size does not change too much with respect to the number and type of adjustment variables in the model. ”

Regarding causal inference, VoE is predominantly relevant as a form of sensitivity analysis (e.g. https://biostats.bepress.com/cgi/viewcontent.cgi?article=1306&context=ucbbiostat) and we do not believe it fits into a singular overall framework.

2.8) Line 85/102-110: Using a baseline with no covariates seems simplistic compared to the modeling used in the published studies. It would be great if the authors included whether or not

their baseline model matched the models explored in the original studies. That is, it doesn't seem fair to mount the comparison in Figure 1B, unless the reader is assured that the baseline model is a fair point of reference.

We agree that a baseline model is simplistic, and we simply selected it because we know many microbiome studies do in fact just use simple, unadjusted models, and also because we wanted one model to compare to in order to make the point that fitting many models versus only one can yield new insights and potentially interesting associations/confounders in a discovery-based setting. In other words, a core aim of our study, which is hopefully now clarified, is to compare the benefits of fitting many models vs just one, and in doing so, choosing a baseline for reference seemed to be a reasonable method for doing so.

Further, we feel strongly, as we write in point 2.1, that fitting simply a single baseline model and not mimicking the various study's approaches exactly precludes us from claiming we are wholly testing reproducibility. As we write in other parts of this response, we have worked hard in this latest draft to prevent readers from misinterpreting our intentions.

2.9) Line 93: Provide a citation for the existing database, or use a different word here.

We now reference curatedMetagenomicData, the database in question.

2.10) Line 96: Rheumatoid arthritis and obesity might be additional indications to explore. Related, IBD could be stratified by UC and CD.

We agree completely, and we hope in the future to do systematic deep dives on single diseases. However, in this case, we are limited by the data we have (CMD link), which is focused on these 6 diseases and does not stratify IBD. If the Reviewers feels this to be a substantial weakness barring publication, we are happy to find more data and carry out another literature review, however, given our focus on VoE's application to the microbiome, we feel that adding more diseases contains slightly less marginal benefit than the other analyses we carried out (e.g. exploration of the number of models on observed VoE).

2.11) Line 112: Provide more detail about the models in terms of parametric forms, outcomes, and covariates.

We now write the following, additionally pointing the reader to Supp Table 1, which contains details on this information:

"We next executed a systematic VoE analysis, fitting a total of 6,035,110 models, each employing multiple linear regression with microbial feature abundances as the dependent variable (**Supp Table 1** contains information on the number and type of covariates per disease)."

2.12) Lines 119-130: Figures 1B and 1G do not seem dissimilar, yet the authors state that VoE helped recover reported results lost by the baseline model. The purpose of the baseline model as a comparison merits discussion here. In Figure 1G, might it be better to report the proportion of findings matched, rather than the median association from all models generated?

We have substantially reworked this Figure and our justification of a baseline model (see comment 2.8), merging 1B and 1G and focusing more on recovery/stratification of associations by this study vs. the literature, and less on what could be construed as measuring reproducibility. Figure 2 now contains these data as well as a standalone figure regarding the fraction of matching associations, which we strong agree is a better way to look at VoE than the median alone. We report our new findings in the results and reproduce them, as well as our new Figure 2 + its legend, here:

“We next aimed to stratify associations by their 1) presence in the literature and 2) robustness/recovery as a function of vibration effects. A total of 509 features were FDR significant at baseline, additionally during VoE, and not reported in the literature review (**Fig 2A, blue column)**. 187 were FDR significant at baseline and reported in the literature (**Fig 2A, orange column**). An additional 61 of others (10% of all literature-based associations) found in the literature were FDR significant in at least one vibration but not in the baseline model (**Fig 2A, red columns**). This brought the total number of taxa-disease associations we were able to recover to 248. Additionally, 264 taxa were not found in the literature but were FDR significant at least once during a vibration (**Fig 2A, green columns**). In other words, we observed that modeling VoE was able to both shed light on associations that would be potentially overlooked by single modeling strategies, in some cases recovering results reported in the literature.

As another measure of robustness, for each taxon for each disease, we report the fraction of associations with signs matching the literature (**Fig 2B**). For all diseases except T1D, these distributions of the fraction of associations whose signs matched what was reported before was bimodal. The mode of the distribution was closer to 1, indicating a large frequency of high concordance associations and a moderate frequency of extremely low concordance (i.e. almost all models pointing the opposite direction as the literature) associations. Given the distribution of the data in Fig 2B, we defined a low concordance association as agreeing in direction in 50% of models fit. 27.9% of all features fit into this category; in other words, 1 in 3 features were discordant with the literature in 50% of all vibrations.”

We reproduce the following relevant Figures below with their legends:

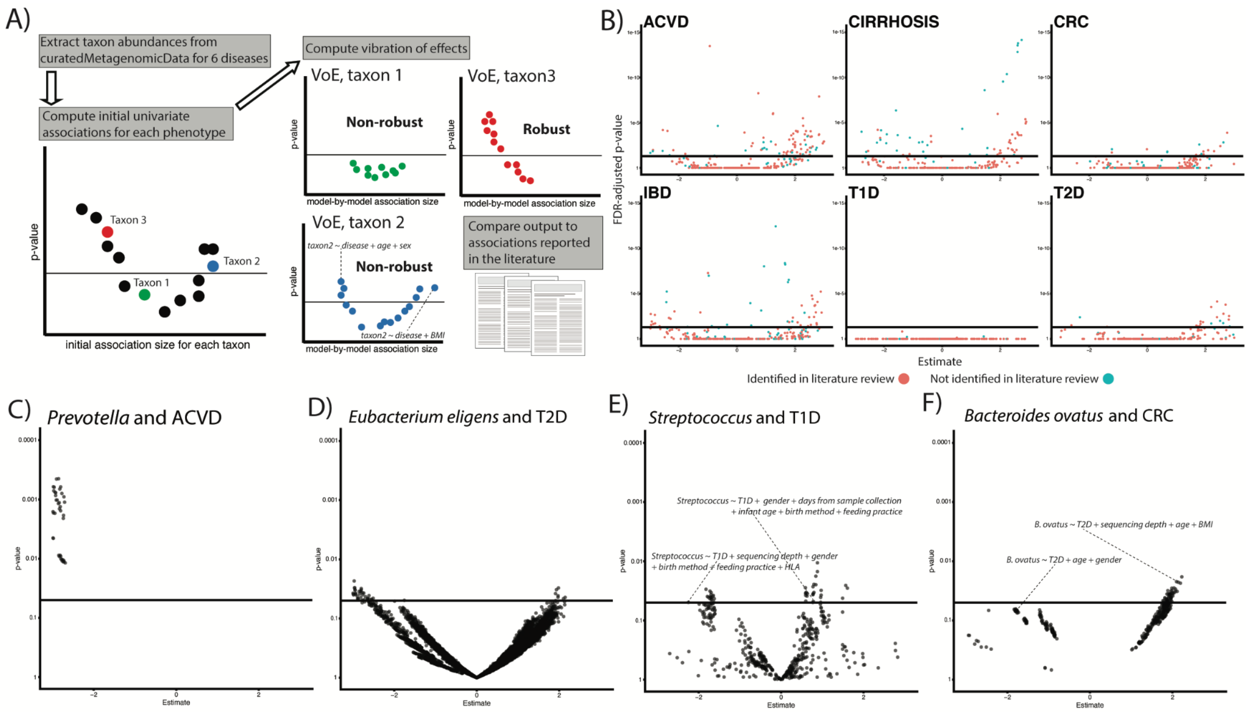

Figure 1: A) Overview of approach. We extract prevalent microbial features from our datasets and attempt to reproduce the findings from the literature by modeling vibration of effects. We additionally review the literature for reported gut microbiome associations (their reported direction of correlation) with six diseases of interest. B) Volcano plots showing the output from the initial, univariate associations. Point color corresponds to if an association was identified in our literature review solid line represents FDR significance (adjusted p < 0.05). C-F) Robust (C) and non-robust associations. Each point represents a different modeling strategy. Solid line is nominal (p < 0.05) significance.

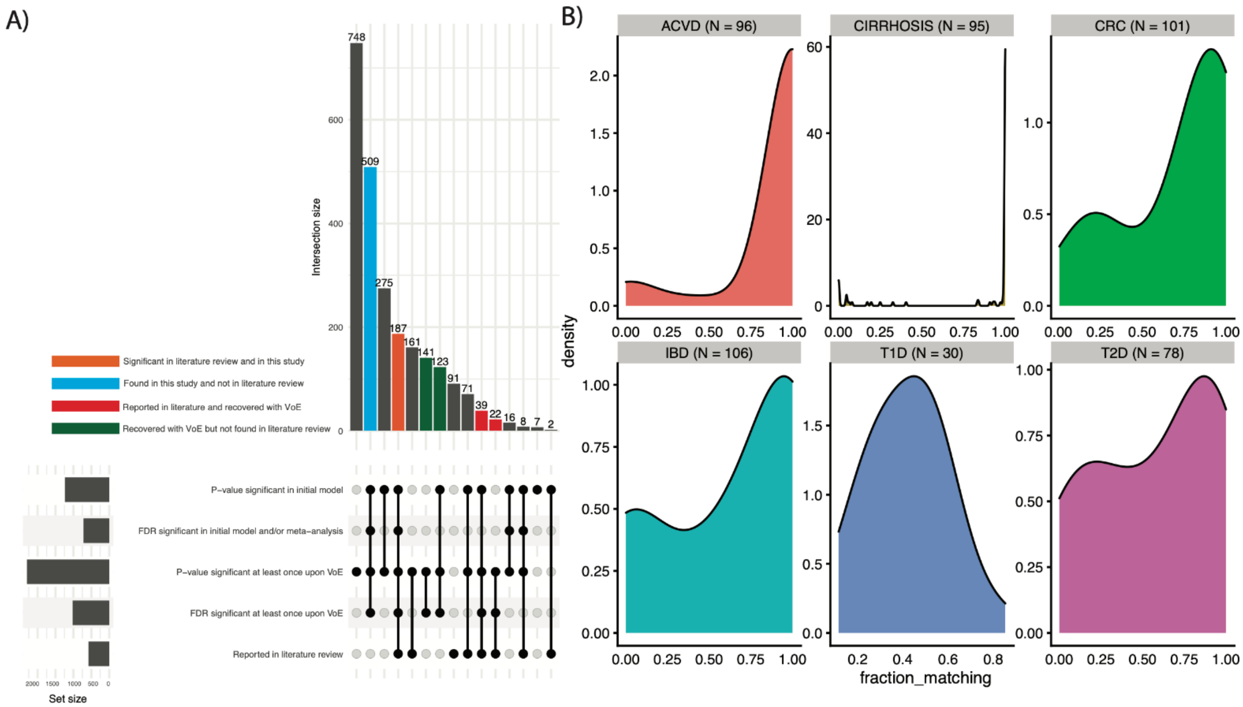

Figure 2: Comparing single modeling approaches to modeling VoE. A) Stratifying features by their being identified in our literature review, p-value/FDR significant (< 0.05) in our initial analysis, or having at least 1 significant model upon vibrations. B) Distribution of fraction of models matching literature-review-defined direction of associations.

2.13) Line 131: Could association robustness also be varying as a function of number of adjusters, rather than disease/cohort? T1D and T2D are reported to be the least robust, but these studies also resulted in the most models generated due to their high number of available covariates.

We agree that VoE is contingent on the number of adjusters, and we believe we have addressed this in points 2.1 and 2.3 with increased discussion as well as our subsampling analysis across diseases.

2.14) Line 148: What is an arbitrary model? It is not clear if this statement refers to a null distribution or probability under the empirical distribution or something else?

We agree this was unclear, and arbitrary was not a fair word to use. We simply meant to refer to a model (akin to our baseline model) where adjusting variables were either not included nor not considered thoughtfully (e.g., in a direct acyclic graph). We believe careless model specification can lead to perplexing results, such as the story of the confounding effect of metformin on type 2 diabetes - microbiome associations, which took many years to untangle.

To avoid having to define an "arbitrary" model, we have removed the word "arbitrary" from this point in the manuscript to shift reader focus to the issue of fitting one, instead of many, models.

2.15) Line 150/296: Figure 2 does not have an A and B panel. Also, is the baseline comparison necessary to include?

The inclusion of 2A was in reference to a prior draft of that figure and erroneous. We have addressed this issue in the text We would prefer to include the baseline comparison to highlight the difference between fitting one model and fitting many. We now mention this justification in the text (see point 2.8), however if the Reviewer's feel strongly about this point we are of course happy to remove it and comply.

Here is the reproduced Figure 2 (now 3) legend with the references to panel A removed:

"**Figure 3**: VoE for reported associations from the literature in the form of summarized modeling output Red blocks indicate organisms that were FDR significant in our study. The middle bar describes the fraction of association sizes greater than 0 per taxon: a highly confounded association will be closer to 0.5 and pink, whereas more robust associations will be closer to 0 or 1 and green. The grey bars in the upper barplot corresponds the fraction of models that were nominally (p-value < 0.05) significant for the microbial feature-disease association, whereas the black bars correspond to the fraction of models that were FDR significant. Features marked as significant in our study but never FDR significant were only significant after the meta-analysis and did not have any nominal significant p-values. See the supplemental figures for this plot reproduced with species names on the x-axis."

2.16) Line 157-159: It would be helpful to explain why the marginal association is not significant.

We now have moved discussion of this result to the Discussion and we have attempted to explain why it was not significant by identifying that none of the non-significant models were adjusted for gender. We write:

"...the association sizes all pointed in the same direction, and 74/127 (58.6%) of models were FDR-significant, despite the univariate association being not (**Supp Fig 3**). We examined the variables present in each model and identified that 64/74 of the FDR-significant vibrations were adjusted for gender, whereas none of the non-significant models were."

2.17) Lines 155-161/337/403: The previously reported association between Roseburia and ACVD was found via an ACVD-microbiome association based on gene and KEGG content, rather than taxa. We wonder if the writers of this manuscript have considered exploring more than relative abundance of species. Gene content and pathway presence/absence could also be explored. Related, because this is already a known association, we are assuming the authors believe this to be potentially overlooked based on their baseline model in this work.

We absolutely agree that this work could be expanded to look at gene and pathway content. Indeed, there are so many ways to slice these kinds of studies -- different transformations of data, different data types, different model specification, so as we write in point 2.1/the

discussion, it is for this reason we attempted to avoid talking too much about “reproducibility.” There is always something else to test, and so instead of trying to go through everything, we simply aimed to focus on VoE with a single data type here. In other manuscripts (https://journals.plos.org/ploscompbiol/article?id=10.1371/journal.pcbi.1007895, https://www.nature.com/articles/s41467-021-23029-8) we exerted effort to compare data types, and as the Reviewers predicted, species are certainly not universally the best way to explore microbiome data, just one lens with a number of flaws.

2.18) Lines 162-192: The authors present the influence of adjuster variables on these particular datasets, but they do not show that these can be prioritized generally in a so-called association-prioritization framework across multiple studies and diseases.

We thank the Reviewer for this comment, and we have attempted to address it in point 2.4, stating that VoE is but one aspect of forming a potential association-prioritization framework.

2.19) Lines 201-203: Is this a reference to confounders, colliders, or something else?

We now specify that we were referring to colliders.

2.20) Lines 226-228: We disagree that an association-prioritization framework has been provided, but maybe VoE could be applied in the future to do so. In this work, there do not seem to be adjusters that are consistently significant across multiple diseases and studies.

We agree and believe we have addressed this comment in point 2.4. We do not claim in this manuscript draft that we have provided an association-prioritization framework.

2.21) Line 245: Geography didn't seem significant for F. prausnitzii associations.

We agree and have remedied our description in the text.

2.22) Line 256/405: It might make sense to pick one time point from longitudinal studies, rather than averaging.

We agree, and we now reference this in the discussion. This is yet another way modeling strategies could be different, and we feel that testing every dimension of the modeling decision-making process is not possible in a single manuscript. We would rather have fit linear mixed models above all else, however at present the quantvoe package does not have a mixed modeling pipeline due to scaling and convergence issues. We hope to include one in the future, and opted here to average across samples for lack of a better way to adjust out intra-individual variation.

As a result, to address this point, at present we write:

“Furthermore, an ostensibly robust association viewed only through the lens of VoE still could be a false positive or dependent on, for example, data processing pipeline choice (e.g. the decision to average repeated measures data vs. selecting one sample per individual).”

2.23) Line 258/403: We recommend performing CLR before comparing abundances across multiple studies of the same disease.

This is another excellent example of an analytic method that could potentially change output. Logging with a fudge factor is only one way to go about transforming data (and has been reported in the microbiome literature: https://www.nature.com/articles/s41591-019-0406-6), and CLR transformations have their strengths as well. To compare these, we now compute CLR transformations for each of our diseases of interest and compare the output to the 1) logged data and 2) non-logged data.

As expected, the different transformations yielded different output, with CLR and non-logged data yielding the least conservative results. We report these results in the text and include new figures in the supplement (reproduced in point 2.1). We chose to continue forward to the logged datasets as it reflects approaches we and others have taken in past work and it was the most successful at recovering any associations from the literature. We believe this choice is further justified by the fact that a focus of this paper is to recover associations as a function of modeling strategy.

2.24) Line 311: We believe the authors meant to say "y-axis."

We did, thank you for catching this.

2.25) Line 312: Figure 3 does not have colors for adjusters used across multiple diseases, as written.

We have addressed this, removing the sentence in question.

2.26) Line 325: Needs a y-axis label

This figure is now in the main text as part of F1, and it has axis labels.

2.27) Line 371: What is the justification for using the second publication?

This was an example of a difficulty arising from our literature review strategy. We wanted to record the association direction for each feature, however, we felt it was misleading if we happened upon -- by chance -- another publication with conflicting results and chose not to report it.

That said, we believe that our reframing of the manuscript in light of Reviewer comments makes this a much smaller issue, as we are focusing less on the “reproducible/literature review aspect” and focusing even more on “number of significant associations post-vibration.”

2.28) Line 402: We expected the models to be set up as disease~microbial feature + covariates. What is the justification for making the microbial feature the outcome?

This is a point we considered deeply when designing our study. Taking together the difficulty in pinning down causal direction in microbiome studies (i.e. does disease change microbial abundance or vice versa) as well as the fact that these linear models are generally interpreted as causal, we feel having the model design could justifiably either be disease ~ microbial_feature or microbial_feature ~ disease. Our decision was made more difficult by the fact that we’d seen both approaches taken in the literature.

Knowing that either approach could be supported and that interpretation could be cloudy regardless, we opted to take what we viewed as the simpler modeling strategy (linear instead of logistic regression), aware that we had used it in prior publications to reviewer satisfaction.

2.29) Line 428: 10,000 is a lot of models. This data set could be used to explore how the number of covariates affects the number of robust associations. What happens if a random 10, 100, 1000 models are used? Or if all models for a random subset of covariates are used?

We agree and have carried out this type of analysis for each disease. First, though, please note that in the manuscript presenting the package used in this paper published while we were completing these revisions, we do more benchmarking of vibration of effects in the form of downsampling to compare how the number of vibrations affects model output. This analysis is contained in Figure 5C of that manuscript (https://journals.plos.org/plosbiology/article?id=10.1371/journal.pbio.3001398). This analysis was how we got to the maximum number of vibrations being 10,000 -- however, that number of course will vary depending on the cohort.

For the sake of completeness, we repeated this analysis for T2D and report it in Supp Fig 4. We showed that many fewer than 10,000 vibrations are needed to model VoE effectively, which we evaluated as the correlation between the beta coefficients from the confounder analysis on the covariates at different number of vibrations. In other words, we evaluated how many models it took for a given variable’s affect on the key association of interest (disease and microbe) to become consistent.

We reference this in the results:

“As a form of benchmarking, we estimated in the T2D cohort how the beta coefficients on the adjusting variables from the mixed modeling approach changed as a function of the number of vibrations executed (Supp Fig 4). We found 10,0000 vibrations (our upper limit) to be sufficient to identify consistent correlation between these beta-coefficients (pearson >.9).”

And reproduce the figure and legend here:

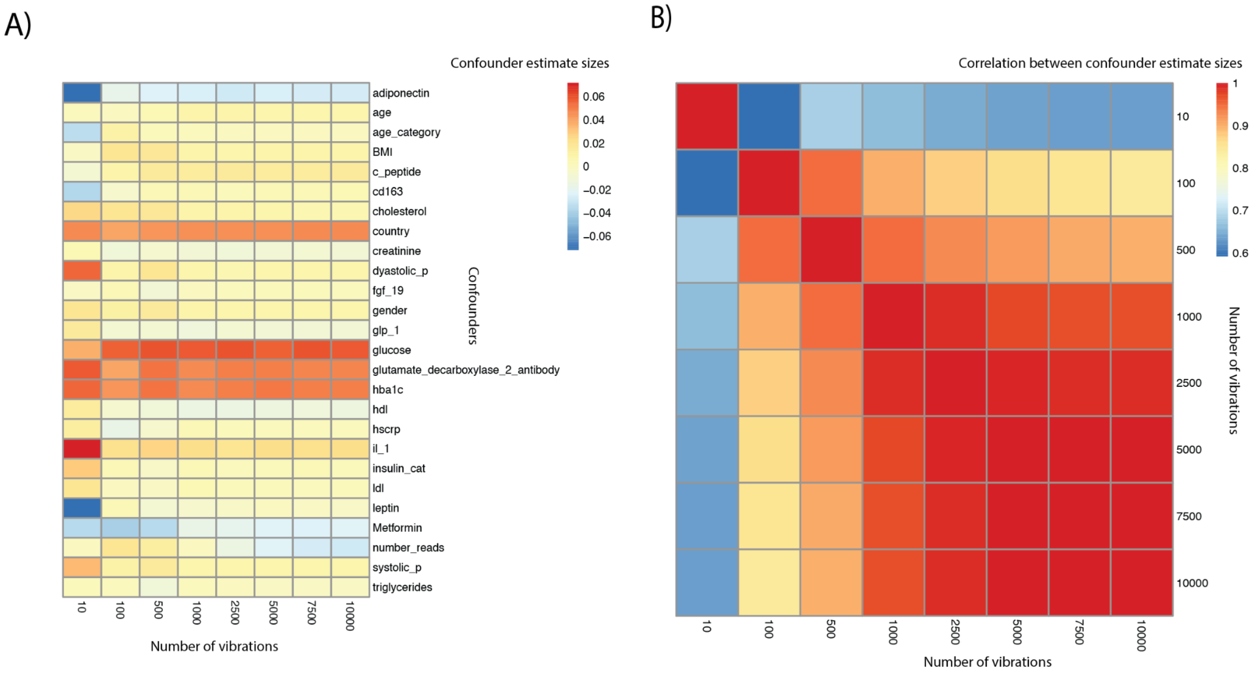

Supplementary Figure 4: Benchmarking the number of vibrations needed to estimate the effect of confounding on microbiome associations. A) The output of our confounder analysis (e.g. in Fig 4). The x-axis is the number of vibrations. The y-axis is each possible adjusting variable in the T2D associations. The values correspond to the beta-coefficient (from our mixed effects analysis) describing the average change in microbiome-disease associations when a given adjusting variable is present in a model. B) The correlation between the values in panel A at different numbers of vibrations.

Style comments:

2.30) In general, there is overuse of quotation marks throughout the manuscript that do not enhance understanding. Examples: "consistent" on line 25, "non-robust" on line 83, "recovered" on line 84, etc.

We have reduce our admittedly excessive use of quotation marks.

2.31) Line 59-62: This is a run-on sentence that hinders understanding.

We have fixed this, thank you.

2.32) Line 86: The phrase after the colon is missing words.

We have removed this paragraph section per other reviewer recommendations.
