# Supplementary material for: Systematically assessing microbiome–disease associations identifies drivers of inconsistency in metagenomic research
Source: PLoS Biol. 2022 Mar 2;20(3):e3001556. doi: 10.1371/journal.pbio.3001556 (PMC8890741; doi:10.1371/journal.pbio.3001556)
Supplement: Attachment — Submitted filename: robustness_response_to_reviewers2.pdf [file pbio.3001556.s009.pdf]

To the Editors and Reviewers,

Thank you for your further consideration of our manuscript.

We have responded once again to the comments of the editor and the Reviewer(s) #2 and hope these changes are satisfactory. As before, our comments are below in blue text with key points highlighted in yellow.

Again, thank you for your time, and we look forward to your response.

Sincerely, and on behalf of the authors,

Chirag J Patel and Aleksandar D Kostic

Editor comments:

a) Please attend to the remaining requests from reviewer(s) #2.

We have now attempted to address the remaining comments from R2.

b) Please re-name your supplementary Fig files "S1_Fig," "S2_Fig," etc.

We have done this.

c) Please address my Data Policy requests below; specifically, we need you to supply the numerical values underlying Figs 1BCDEF, 2AB, 3, 4ABCDEFGH, 5ABC, S1ABCD, S2, S3, S4AB. If these can all be generated from the data and code deposited in Github/Figshare, please cite the location of the data clearly in each relevant main and supplementary Fig legend, e.g. “This Figure can be generated using the data and code deposited in https://github.com/chiragjp/ubiome_reproducibility”).

We have appended this information to each Figure legend, as they all can be reproduced with the data on Figshare and the code on GitHub. Specifically, at the bottom of each Figure legend, we write:

“This Figure can be generated using the code deposited in https://github.com/chiragjp/ubiome_robustness and the data deposited in https://figshare.com/projects/Microbiome_robustness/127607””

Reviewer #2:
[identify themselves as Annamarie Bustion and Katie Pollard]

The paper is improved; the results are presented more clearly and the conclusions drawn are more metered. The authors re-identified their research goals as the following: identification of

confounders, recovery and robustness assessments of previous literature findings, and identification of new associations. The text answers these goals, and the authors appropriately de-emphasized their method's ability to assess reproducibility or provide an umbrella feature prioritization framework. Also, the authors present the rationale for using VoE more comprehensively, and they better describe their rationale for using a simple baseline model rather than recapitulating the models from their literature findings.

We are glad the reviewers found our paper to be improved and thank them for their comments.

Remaining critiques:

Authors' response to 2.1: The authors now provide an assessment of VoE sensitivity to number of variables available, using T2D as an example. This does provide confidence that VoE can provide information on non-robust associations independent of number of variables. However, a stronger response would have looked at larger values for the number of variables, not just a comparison of 3, 6, and 9 variables. Further, an assessment of only three variable sizes seems insufficient to state that there could be a "a potential leveling-off with increasing adjusting variables." We recommend adding results for some higher numbers of variables.

First, we agree with the Reviewers that the “leveling off” comment in the Supplemental Text could use more justification. In short, however, we do not believe this is the dataset to do it due to a limited number of total adjusters being reported in the cohort. As a result, we have removed this claim from the text (in addition to carrying out some of the requested analysis, described below).

The analysis in our initial response was centered on the number of significant findings yielded by different numbers of adjusting variables, transformation methods change, and our single cohort and meta-analytic modeling strategies. Because we were meta-analyzing and wanted to keep the number of variables consistent across cohorts, we selected 3, 6, and 9, variables as our adjusters. The cohort with the least number of adjusters had only 9, therefore we opted not to test higher values for the sake of consistency.

Therefore, in the analysis we executed for this point, we did not meta-analyze. We instead took the cohort with the highest number of adjusters (24) and computed the number of significant findings specifically in this cohort as a function of modeling strategy (for the sake of simplicity, we only considered one data transformation strategy, natural logging). We note that, in this one cohort, the number of adjusters did not appear to have a substantial impact on the FDRsignificant features. We reproduce the new figure and Supplemental Text below.

“That said, between vibrating over 3, 6, and 9 variables (the number of variables in the cohort with the fewest adjusters), the number of potentially significant features for meta-analyzed, log-transformed datasets went from 19 to 30 to 31.”

“We additionally executed a similar, non-meta-analytic, analysis in the T2D cohort with the largest number of potential adjusters (24 variables, Supp Fig 1D-E). We observed consistency in the number of FDR significant variables and those that were p-value significant at least once, for the most part, across different numbers of adjusters. There was some variation however, with the number of ostensibly significant features (especially in the p-value-significant-once category, Supp Fig 1D) slightly increasing as a function of number of adjusters, indicating the potential for excessive vibrations and reliance on p-values alone to yield increased false positives (or, conversely, at the very least, more associations worth investigating).”

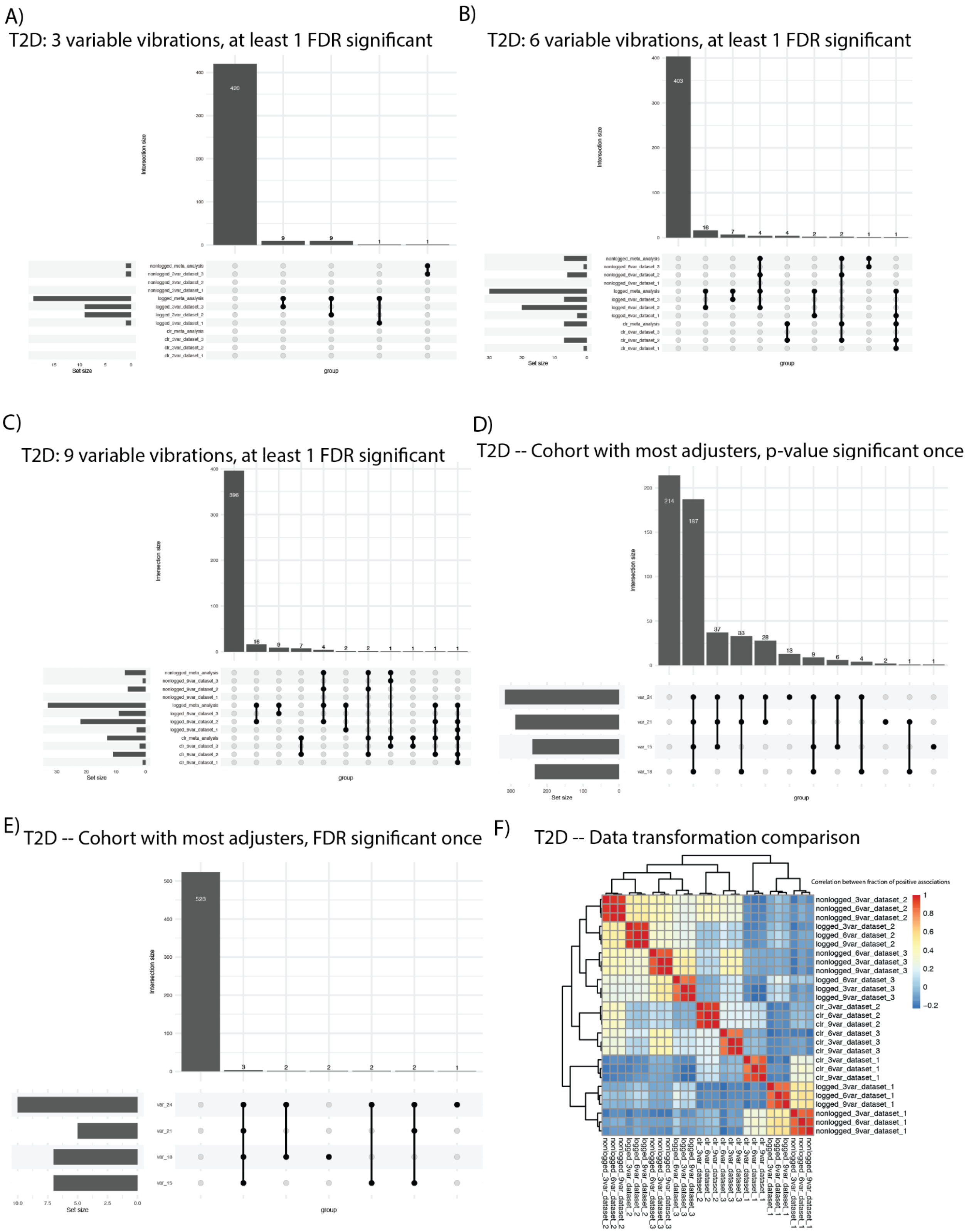

Supplementary Figure 1: Benchmarking data transformations and the number of adjusting variables using our T2D datasets. A-C) The impact of the different numbers of vibrations and data transformation methods on vibration of effects. We plot the number of features that were

FDR-significant at least once upon vibration with different numbers of adjusting variables considered as well as different data transformation strategies (i.e. logged vs raw abundances vs center log-ratio transformations). D) The number of p-value and E) FDR-significant findings for the T2D cohort with the largest number of possible adjusters (using only log-transformed data, as opposed to the previous three panels). F) The robustness of associations as a function of number of vibration variables and modeling strategy. We computed the fraction of associations that were positive for any given microbial feature -- a highly robust association is 100% positive or 0% positive (i.e. negative), whereas a non-robust association is closer to 50% positive (i.e. inconsistent in direction). In this heatmap, we correlated these associations for all features to gauge if the different data transformations and numbers of adjusting variables yielded similar measures of robustness across all datasets. This Figure can be generated using the code deposited in https://github.com/chiragjp/ubiome_robustness and the data deposited in https://figshare.com/projects/Microbiome_robustness/127607"

Authors' response to 2.2: It is now clear how the authors made use of multiple cohorts. But it would have been useful for the authors to instead use additional disease cohorts as a means of external validation in addition to the use of previous literature findings. If possible, we recommend adding this analysis.

We agree that validation in external cohorts would absolutely be ideal. However, we claim that it is outside the scope of this particular study. We are not necessarily trying to report new associations here (as we were in this companion paper that also uses VoE, where we did do external validation: https://www.nature.com/articles/s41467-021-23029-8). Given that the focus of this manuscript was looking at VoE in cohorts within curatedMetagenomicData, we think that adding in more public data would not necessarily underscore the points within the paper already. Indeed, such an analysis is likely worthy of another manuscript on its own.

We hope this will be acceptable to the Reviewers and Editors.

Abstract: Before reading the main text, it is not clear what "model specifications" refers to. This could be different covariates, a different parametric form, or a data transformation, amongst other things. It would be very helpful to clearly state that the focus of this paper is exploring all the possible combinations of covariates. Note: In the Introduction (L74-76), this is pretty clear. Our recommendation is to include a similar definition / scope statement in the abstract.

We now write the following in the abstract:

"Systematically exploring different combinations of adjusting covariates in modeling approaches (i.e. model specifications) revealed associations that could potentially be overlooked when restricting analyses to one or a few modeling strategies."

Abstract/Introduction: We appreciate that the authors now also look at non-associations with VoE. We recommend that you check the places where you talk about focusing on 581 published

associations: some of the text sounds like you only analyze the associations but not the non-associations. Alternative language might be "studies" or "cohorts" "in which disease-microbiome associations were previously detected".

We have now gone through the Abstract and Introduction as requested and updated the language accordingly.

Figure 2A: The provided Upset plot is not fully explained. What do the gray bars represent?

We now write:

"**Figure 2**: Comparing single modeling approaches to modeling VoE. A) Stratifying features by their being identified in our literature review, p-value/FDR-significant (< 0.05) in our initial analysis, or having at least 1 significant model upon vibrations. The gray bars labeled "set size" indicate the number of features associated with a given row in the bottom of the plot (e.g. about 1000 features were p-value significant in the initial model). The gray bars in the upper portion of the panel are those we chose not to highlight, as they do not fall into any category indicated by the colors or referenced in the manuscript. B) Distribution of fraction of models matching literature-review-defined direction of associations."

Figure 2B: These results are more interpretable now that the authors are exploring fraction of matching associations. But these graphs would be more legible as count histograms rather than density plots.

We have now converted these plots to histograms and reproduce the relevant Figure below:

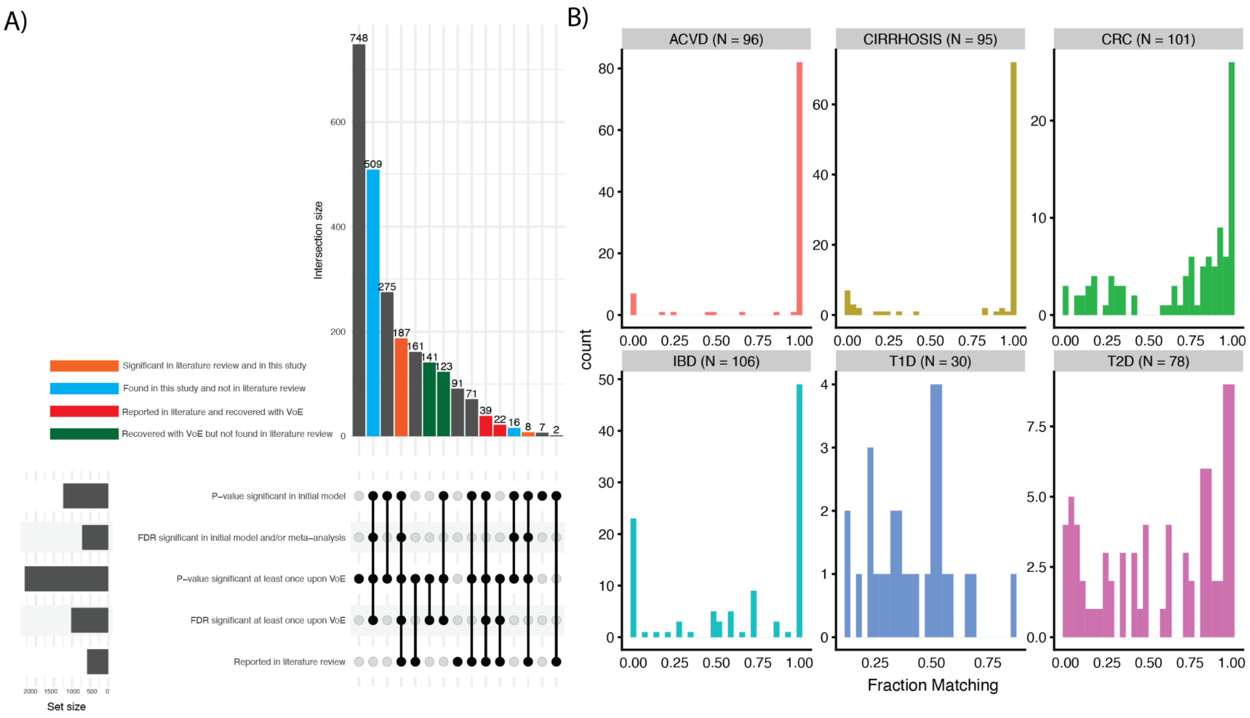

It is our policy to sign reviews: Annamarie Bustion and Katie Pollard
